# Gut microbiota metabolite indole-3-acetic acid maintains intestinal epithelial homeostasis through mucin sulfation

**DOI:** 10.1080/19490976.2024.2377576

**Published:** 2024-07-27

**Authors:** Mengfan Li, Yiyun Ding, Jingge Wei, Yue Dong, Jingyi Wang, Xin Dai, Jing Yan, Feifei Chu, Kexin Zhang, Fanyi Meng, Jiahui Ma, Weilong Zhong, Bangmao Wang, Yunhuan Gao, Rongcun Yang, Xinjuan Liu, Xiaomin Su, Hailong Cao

**Affiliations:** a Department of Gastroenterology and Hepatology, Tianjin Medical University General Hospital, National Key Clinical Specialty, Tianjin Institute of Digestive Diseases, Tianjin Key Laboratory of Digestive Diseases, Tianjin, China; b Department of Immunology, Nankai University School of Medicine, Nankai University, Tianjin, China; c Department of Gastroenterology, Beijing Chaoyang Hospital, Capital Medical University, Beijing, China

**Keywords:** Inflammatory bowel disease, indole-3-acetic acid, aryl hydrocarbon receptor, mucin, sulfation

## Abstract

The global incidence and prevalence of inflammatory bowel disease (IBD) are gradually increasing. A high-fat diet (HFD) is known to disrupt intestinal homeostasis and aggravate IBD, yet the underlying mechanisms remain largely undefined. Here, a positive correlation between dietary fat intake and disease severity in both IBD patients and murine colitis models is observed. A HFD induces a significant decrease in indole-3-acetic acid (IAA) and leads to intestinal barrier damage. Furthermore, IAA supplementation enhances intestinal mucin sulfation and effectively alleviates colitis. Mechanistically, IAA upregulates key molecules involved in mucin sulfation, including 3’-phosphoadenosine 5’-phosphosulfate synthase 2 (Papss2) and solute carrier family 35 member B3 (Slc35b3), the synthesis enzyme and the transferase of 3’-phosphoadenosine-5’-phosphosulfate (PAPS), via the aryl hydrocarbon receptor (AHR). More importantly, AHR can directly bind to the transcription start site of Papss2. Oral administration of *Lactobacillus reuteri*, which can produce IAA, contributes to protecting against colitis and promoting mucin sulfation, while the modified *L. reuteri* strain lacking the *iaaM* gene (*Lactobacillus*^*ΔiaaM*^) and the ability to produce IAA fail to exhibit such effects. Overall, IAA enhances intestinal mucin sulfation through the AHR-Papss2-Slc35b3 pathway, contributing to the protection of intestinal homfeostasis.

## DESCRIPTION OF GRAPHICAL ABSTRACT

A HFD can lead to the development of colitis by disrupting tryptophan metabolism in the gut microbiome and lowering levels of IAA. Supplementation with IAA has been shown to alleviate colitis in mice and improve intestinal barrier function. It is believed that IAA may activate the AHR to upregulate the expression of Papss2 and Slc35b3, promoting sulfation modification of mucins and protecting the intestinal barrier. HFD, high-fat diet; AHR, aryl hydrocarbon receptor; IAA, indole-3-acetic acid; Papss2, 3’-phosphoadenosine 5’-phosphosulfate synthase 2; Slc35b3, solute carrier family 35 member B3.

## GRAPHICAL ABSTRACT

**Figure:**
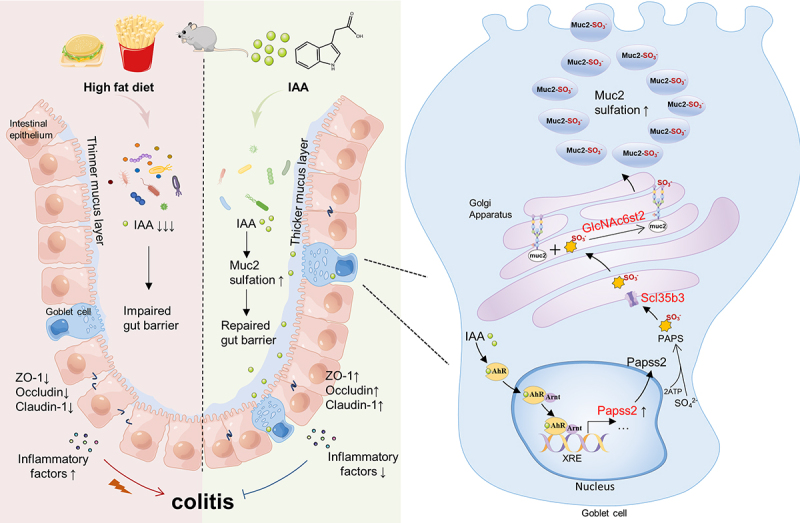

## 1. Introduction

Inflammatory bowel disease (IBD) has exhibited a global increase in prevalence in recent decades, yet its etiology remains elusive.^1,2^ The onset of IBD is related to environmental factors (such as a Western-style diet), genetic susceptibility, disturbances in the composition of the intestinal microbiota and compromised intestinal barrier integrity.^3^ Metagenomics and metabolomic analysis have revealed significant dysbiosis in the intestinal microbiota and their metabolites in IBD patients, characterized by decreased species diversity, reduced abundance of *Firmicutes*, and an increase in *Enterobacteriaceae*. Metabolites of the gut microbiota, such as short-chain fatty acids, secondary bile acids, and tryptophan derivatives, are diminished.^4,5^ Epidemiological research indicates a synchronicity between the rise of Western-style diets globally and the regional increase in IBD incidence and a Western-style diet is significantly correlated with heightened risks of IBD progression.^6^ A soybean oil-based high-fat diet (HFD) increases susceptibility to colitis in IL-10 knockout mice via reduction in anti-inflammatory microbial metabolites,^7^ and a 45% fat diet, in conjunction with antibiotics, synergistically impairs mitochondrial function in the intestinal epithelium, precipitating dysbiosis of the gut microbiota and exacerbating mucosal inflammation.^8^ However, the links between exposure to a HFD and the disorder in intestinal homeostasis remain incompletely understood.

Tryptophan, an essential aromatic amino acid in the human body, can be further processed by the gut microbiota within the gut. Microbial-derived tryptophan metabolites, such as indole, indole-3-aldehyde (I3A), indole-acetaldehyde, and indole-lactic acid (ILA), have been recognized for their significant impact on host physiology by acting as ligands for the aryl hydrocarbon receptor (AHR).^9–11^ AHR is a widely conserved ligand-activated transcription factor and resides in the cytoplasm in the absence of ligand activation. Upon ligand binding, it translocates into the nucleus and form a complex with the AHR nuclear translocator protein (Arnt). This complex then binds to xenobiotic response elements (XREs) on DNA, thereby regulating the transcription of target genes.^12,13^ ILA, generated by bacteria like *Escherichia coli*, downregulates epithelial CCL2/7 production through AHR, thereby mitigating macrophage activation.^14^ Moreover, I3A, indole-3-pyruvate, and indole-3-ethanol, three tryptophan metabolites originating from gut microbes, exhibit potential in preserving intestinal epithelial barrier integrity during dextran sulfate sodium (DSS)-induced colitis.^9^ Nevertheless, the impacts and underlying mechanisms of additional tryptophan derivatives in maintaining intestinal homeostasis remain uninvestigated. Clinical evidence suggests that IBD patients exhibit reduced expression and diminished activity of AHR in inflamed tissues, this phenomenon could be related to the reduction of tryptophan metabolites.^15^

The mucus layer, positioned between the large intestinal microbial mass and epithelial and immune cells, plays a vital role in preserving the segregation of luminal contents, including bacteria, from epithelial cells, primarily consists of highly glycosylated mucin 2 (MUC2), which is further divided into acidic and neutral mucins, as well as subtypes of sulfomucin and sialomucin.^16,17^ Sulfomucin represents a prevalent form of O-linked glycan modification, abundant in both the small intestine and colon, with relatively higher levels observed in the distal colon. Augmented levels of sulfated mucins in the distal colon serve to impede bacterial enzymatic degradation of mucin glycans, thereby effectively preventing intimate contact between bacteria, harmful factors, and the intestinal epithelium.^18^

In this study, we observed that a HFD disrupts tryptophan metabolism in the murine gut, leading to a significant decrease in indole-3-acetic acid (IAA) levels. Subsequent supplementation of IAA and *Lactobacillus reuteri* capable of producing IAA resulted in alleviation of colitis in mice and thickening of the colonic mucus layer. Mechanistically, IAA upregulated the expression of key molecules 3’-phosphoadenosine 5’-phosphosulfate synthase 2 (Papss2) and solute carrier family 35 member B3 (Slc35b3) involved in mucin sulfation via AHR, thereby enhancing intestinal barrier function and ameliorating colitis.

## 2. Materials and methods

### 2.1. Dietary survey and human samples collection

Patients diagnosed with ulcerative colitis (UC) or Crohn’s disease (CD) based on clinical presentation, radiological findings, endoscopy, and histopathology were recruited from the General Hospital of Tianjin Medical University, China, between January 2019 and January 2023. The dietary habits of these UC and CD patients were assessed through the administration of a Semi-Quantitative Food Frequency Questionnaire. To calculate daily fat intake (in grams) and total calorie consumption (in kilocalories), the Chinese Food Ingredients List (6th Edition) was used as a reference. Daily fat intake was expressed as fat intake (g) × 9 (kcal/g)/total calorie intake (kcal). Concurrently, clinical disease severity indicators, including Mayo clinical scores, erythrocyte sedimentation rate (ESR)，fecal calprotectin, Crohn’s disease activity index, endoscopic images and colonic histopathological images were recorded for each patient. The correlation between daily fat intake and these clinical indicators were assessed through Pearson’s correlation analysis.

The colonic biopsies for Single-Cell RNA sequencing were obtained from four patients who were diagnosed as left-sided moderate UC at the Department of Gastroenterology, Beijing Chaoyang Hospital of Capital Medical University (Beijing, China). Prior to their participation, written informed consent was obtained from the patients, and ethical approval was granted by the Ethics Committee of the Beijing Chaoyang Hospital, Capital Medical University (2018-8-20-2).

The colonic biopsies for section staining were obtained from UC patients at the General Hospital of Tianjin Medical University (Tianjin, China). Written informed consent was obtained, and the ethical approval was granted by the Ethics Committee of Tianjin Medical University General Hospital, China (IRB2020-KY-074). The samples were kept at −80°C after being snap-frozen.

### 2.2. Animals and experiments

6- to 8-week-old female C57BL/6J mice were purchased from Hua Fukang Biotechnology, Beijing, China. *Ahr*^*−/−*^ mice were obtained from the State Key Laboratory of Medicinal Chemical Biology, Nankai University. The mice were co-housed in a specific pathogen free (SPF) room with light and temperature-controlled. After being housed in 12-h light/dark cycles for 7 d, where they had unrestricted access to food and drink, the mice were divided into 4–6 groups and placed in cages.

For the HFD and colitis model, the C57BL/6J mice in the control group were continually fed a standard laboratory chow diet, while mice in the HFD group were fed a HFD (20% protein, 20% carbohydrate and 60% fat) (H10060, Beijing HFK Bioscience CO., LTD). Two weeks later, acute experimental colitis was induced, mice were given 2.5% DSS(0216011080, MP Biomedicals) in their drinking water for 7 d and daily measurements of their body weight and disease activity index (DAI) score were made according to the previously reported methods.^19^ The fresh fecal samples were collected and mice were anesthetized and sacrificed after the experiment. The entire colons were resected and the colon lengths were measured. Portions of the colon were fixed in 4% formaldehyde solution for histopathologic examination, the left colon tissues were reserved in −80°C refrigerators.

For the IAA supplementation and colitis model, the C57BL/6J mice were administered IAA (20 mg/kg, S18031, Yuanye Biotechnology) dissolved in dimethylsulfoxide (DMSO, ST038, Beyotime) via oral gavage every 24 h for 7 d before 2.5% DSS treatment. Control mice received equivalent volumes of DMSO via gavage. As mentioned before, the DAI score and body weight were accessed daily. In vivo examinations of the mice using a small animal colonoscopy system (Shanghai Yuyan Instruments Co., Ltd., China) were performed on day 7 after DSS administration. To assess disease severity, we used the previously established Murine Endoscopic Index of Colitis Severity (MEICS),^20^ which takes into account various criteria including colon thickening, changes in vascular pattern, visibility of fibrin, mucosal surface granularity, and stool consistency. The entire colons were resected and the colon lengths were measured. The distal colons without washing were fixed in Carnoy’s fixative for histopathologic examination, the left colon tissues were sliced into tiny fragments and reserved in − 80°C refrigerators.

To evaluate the therapeutic potential of IAA in colitis and to mitigate any potential interference of IAA with the pharmacological effects of DSS, we initially administered drinking water containing 2.5% DSS to mice for 7 d. Subsequently, the mice were transitioned to sterile drinking water while concurrently receiving a daily oral gavage of 20 mg/kg IAA for 5 d. Body weight and DAI score were measured daily as previously described. Upon completion of the experiment, the mice were sacrificed, and the entire colons were removed and measured. The colons were then fixed in Carnoy’s fixative solution for histopathological examination.

For 2,4,6-trinitrobenzene sulfonic acid (TNBS, P2297, Sigma-Aldrich)-induced colitis model, the C57BL/6J mice were separated into four groups randomly: vehicle, IAA, TNBS, and TNBS+IAA groups. The IAA group and TNBS+IAA group were pre-administered IAA 7 din advance, while the vehicle group and TNBS group were administered DMSO as a control. On the 8th day, the mice in TNBS and TNBS+IAA groups were disposed intrarectally with 150 μl of 2.2% TNBS solution in 50% ethanol as experimental group, mice in vehicle and IAA groups received 150 µl of 50% ethanol enema as controls, and concurrently, IAA or DMSO was continued to be administered to the mice for the following 1–3 d. The survival rate and change in body weight were noted every day. On the 11th day, mice were slaughtered while under anesthesia. The colon lengths were measured and collected for histopathological analysis.

To establish a model of spontaneous colitis, 10-week-old female IL-10-deficient mice were purchased from GemPharmatech, Jiangsu, China. The mice were housed in an SPF environment as previously described. After a one-week acclimation period, they were divided into two groups, which received equal doses of 5% DMSO and IAA (20 mg/kg) dissolved in 5% DMSO, respectively, for a duration of 37 d. Body weight and DAI score were measured daily as mentioned before. Upon completion of the experiment, the mice were sacrificed, and the entire colons were removed and measured. A 0.5 cm segment each from the proximal, mid, and distal colon was fixed in Carnoy’s fixative solution for histopathological examination. The remaining portions of the left colons were sliced and stored at −80°C.

For the bacteria administration and colitis model,the C57BL/6J mice were administered an antibiotic cocktail (ABX) with ampicillin (A, 1 g/l), metronidazole (M, 1 g/l), neomycin sulfate (N, 1 g/l), and vancomycin (V, 0.5 g/l) through their drinking water containing 0.1% sucrose for a duration of two weeks. Every 3 d, the antibiotic-containing water was switched out. After 2 weeks of treatment, the mice were switched to sterile tap water for 2 d. The mice were then divided into 3 groups: PBS, *L. reuteri* (*Reu*.) and *Lactobacillus*^*ΔiaaM*^ (*△iaaM Reu*.) treatment groups. Bacteria were administered via oral gavage twice a week at a dosage of 1 × 10^9^ CFU per administration. 2 weeks later, for a period of 12 d, 2.5% DSS was added to the drinking water to induce acute experimental colitis. Daily measurements were taken for body weight and DAI. After removing each colon completely, lengths were measured. Portions of the colon were fixed in 4% formaldehyde solution for histopathologic examination.

The institutional Animal Care and Use Committee of the Tianjin Medical University, Tianjin, China (TMUaMEC2022025), approved all animal housing and experimentation procedures and ensured that they followed ethical guidelines

### 2.3. Cell culture and treatment

HT29 cells were acquired from ATCC and cultured in Roswell Park Memorial Institute 1640 (PM150110, Procell) supplemented with 10% Seradigm Premium Grade Fetal Bovine Serum (FBS 164,210–50, Procell), 1% non-essential amino acids (PB180424, Procell), and 1% penicillin/streptomycin (PB180120, Procell). LS174T cells were obtained from Procell Life Science&Technology and grown in Minimum Essential Medium supplemented with 1% non-essential amino acids, 1% penicillin/streptomycin and 10% FBS. Both of them were grown at 5% CO_2_ and 37°C. A 12-well plate was seeded with 5 × 10^6^ cells per well. The cells were subsequently treated for 24 h with IAA (100 μM), lipopolysaccharide (LPS, 100 ng/ml, ST1470, Beyotime), or IAA + LPS with or without an AHR inhibitor (10 μM, CH223191, HY-12684, MedChemExpress) or PXR inhibitor (10 μM, Resveratrol, HY-16561, MedChemExpress).

### 2.4. LC-MS/MS

The solid samples were added to the extract solution (methanol: acetonitrile: H2O = 2:2:1) and vortexed for 30 s. Then, they underwent a 4-min grinding at 35 Hz and a 5-min ultrasonication in an ice-water bath. The above steps were repeated twice and followed by stabilizing at −40°C for 1 h. After centrifugation at 4°C 12,000 rpm for 15 min, the supernatant was separated and dried with nitrogen, and then 0.1% formic acid aqueous solution was added for redissolving. After repeated centrifugation at 4°C 12,000 rpm for 15 min, the supernatant was removed in preparation for UHPLC-MS-MS analysis. A typical resolution was prepared and diluted in turn to obtain a series of calibration solutions. Sciex MultiQuant (Version 3.0.3) and CIEX Analyst Work Station (Version 1.6.3) were used to collect and process MRM data.

### 2.5. RNA sequencing

Following the collection of colon cells and subsequent PBS wash, the TRIZOL (cat #15596026, Ambion) reagent was used in order to extract total RNA. The enrichment of pathways was investigated using gene set enrichment analysis. To determine up-regulated cellular pathways, hallmark gene sets database was performed to analyze datasets. Member genes with 1,000 permutations were ranked and the top ones were selected to calculate the enrichment score. The minimum gene set size was fixed at 15 genes while the maximum size was 500 genes. We selected the gene sets with the false discovery rate of q < 0.25 for pathway enrichment.

### 2.6. Generation and culture of Lactobacillus^ΔiaaM^

The gene coding for *iaaM* in *L. reuteri* was deleted by utilizing the pNZ5319 plasmid. Initially, after being amplified, the upstream and downstream *iaaM* gene pieces were inserted into the Bgl II, Sac I, Pem I, and Xoh I digestion sites of the pNZ5319 plasmid. Subsequently, competent *L. reuteri* cells were electrotransferred with the recombinant pNZ5319 plasmid. The single-exchange Lactobacillus cells containing the recombinant pNZ5319 plasmid resistant to chloramphenicol were selected and validated using the primers listed in Table 1. Following 40 generations of culturing single-exchange *L. reuteri* cells at 30°C, double-exchange strains were ultimately selected that exhibited resistance to chloramphenicol but not erythromycin. Finally, the deletion of *iaaM* in *L. reuteri* (*Lactobacillus*^*ΔiaaM*^) was confirmed using the primers indicated in Table 1. *Lactobacilli* were cultured in MRS broth and subsequently plated on MRS agar supplemented with 10% sucrose. Using AnaeroPack-Anaero sachets (Mitsubishi Gas Chemical, Japan) within a hermetically sealed jar, anaerobic conditions were reached. To evaluate the production of IAA in vitro, *Lactobacillus reuteri* and *iaaM* deleted *Lactobacillus reuteri* were routinely cultured in MRS broth at 37°C for 24 h. Subsequently, monoclonal *Lactobacillus* was regenerated in MRS broth supplemented with or without 3 mM tryptophan to potentially induce tryptophan catabolism. The supernatant was gathered at the designated moment, and IAA levels were examined by IAA Chemiluminescent Immunoassay Kit (abx190011, Abbexa).

**Table 1.** Primer sequences used for realtime-PCR.

Primers		Sequence
mGAPDH	Forward	5'-GGAGAAACCTGCCAAGTATG-3'
	Reverse	5'-TGGGAGTTGCTGTTGAAGTC-3'
mPapss2	Forward	5'-TGGTGCTGGGAAAACAACCA-3'
	Reverse	5'-TCCCCCGCAGAGAATCCCAG-3'
mSlc35b3	Forward	5'-CTGTGGGTACTATGGGCTTATCA-3'
	Reverse	5'-CATAACAGGAATCAGTTTGCAGC-3'
mGlcNAc6ST2	Forward	5’-TCCATACTAACGCCAGGAACG-3'
	Reverse	5'-TGGTGACTAAGGCTGGAACC-3'
mIL-1β	Forward	5’-ACGGACCCCAAAAGATGAAG-3'
	Reverse	5'-TTCTCCACAGCCACAATGAG-3'
mIL-6	Forward	5’-CCAGTTGCCTTCTTGGGACT-3'
	Reverse	5'- GGTCTGTTGGGAGTGGTATCC-3'
mTNF-α	Forward	5’- CTTCTGTCTACTGAACTTCGGG-3'
	Reverse	5'-CAGGCTTGTCACTCGAATTTTG-3'
hGAPDH	Forward	5'-GGAGAAACCTGCCAAGTATG-3'
	Reverse	5'-TGGGAGTTGCTGTTGAAGTC-3'
hPapss2	Forward	5'-AGACGGAGAACCAGCAGAAAT-3'
	Reverse	5'-CACACGGTACATCCTCGGAAC-3'
hSlc35b3	Forward	5'-TTACTGGGATTGACATGCACTAG-3'
	Reverse	5'-AGCCAGAACAAAGGAGATTCC-3'
hGlcNAc6ST2	Forward	5'-CCTCCCTCAACCTGCATATCG-3'
	Reverse	5'-TCACAATGCGACTGTCAATCA-3'
iaaM	Forward	5'-ATGACCAAAACCAACTATATCAATG-3'
	Reverse	5'-TTTAGCCGCTTGATGAACTTGA-3'
Cre	Forward	5'-CTAACTCGAGTGATCACCAATTC-3'
	Reverse	5'-GGCTATCAATCAAAGCAACACG-3'
CM	Forward	5'-ATGAACTTTAATAAAATTGATTTAGACAATTG-3'
	Reverse	5'-TTATAAAAGCCAGTCATTAGGCCTATC-3'
iaaM-up	Forward	5'-CCGCTCGAGAACGTTTTCCATCAAGTTGAGC-3'
	Reverse	5'-AGCTTTGTTTAAACAACTGACTATTCACCACGCCTC-3'
iaaM-down	Forward	5'-CGAGCTCAATCACACACAATCAACTATGGACA-3'
	Reverse	5'-GAAGATCTGCAAGTCCCGTTTGAACATCT-3'
JS00368-IL10-5 wt-tF1		5'-GCCATGAGTTAAACTAAACCCAGGC-3'
JS00368-IL10-3 wt-tR1		5'-TAGGCATCTCTTGCCTAGTGTTGGT-3'
Bacteria primers		
16s 27F		5’- AGAGTTTGATCCTGGCTCAG-3’
16s 1492 R		5’- GGTTACCTTGTTACGACTT-3’
L.Reuteri-Fs		5'-ACCGAGAACACCGCGTTATTT-3'
L.Reuteri-Rs		5'-CATAACTTAACCTAAACAATCAAAGATTGTCT-3'
iaaM-Fs		5'-GGGGTAAAAGAGGCGGTTCA-3'
iaaM-Rs		5'-GATTACCTTTTCACGCGCCC-3'

### 2.7. Bacterial quantification

Fecal samples were collected, and total DNA was extracted using the DNA Stool Kit (QIAGEN, Germany). Quantitative PCR was then performed to determine the relative abundance of *L. reuteri* and *Lactobacillus*^*ΔiaaM*^, as previously described.^21^ The quantification of *L. reuteri (Reu.)* and *Lactobacillus*^*ΔiaaM*^ (*ΔiaaM Reu*.) was measured relative to the universal bacterial 16S gene, with the primers listed in Supplementary Table 1.

### 2.8. Intestinal permeability measurement

To examine intestinal permeability, mice were fasted for 4 h prior to the assay and were administered 0.6 mg/g of fluorescein isothiocyanate-labeled dextran (MW 4000) (MedChemExpress, HY-128868A) via gavage. Two hours later, serum samples were collected and centrifuged. The supernatant was then diluted and subjected to fluorescence detection (488 nm excitation/525 nm emission). The concentration of FITC-dextran in the samples was determined using a standard curve generated from serial dilutions of FITC-dextran.

### 2.9. Quantitative real-time polymerase chain reaction (qRT-PCR)

Using the RNA isolater Total RNA Extraction Reagent (Vazyme, China), total RNA was isolated from cells and colon tissues. Subsequently, cDNA synthesis was carried out using the HiScript III RT SuperMix for RT-PCR (Vazyme, China) as per the manufacturer’s instructions. 0.08 g/L spermine (S425636, Aladdin) was used to purify total RNA from mouse tissues treated with DSS. The ChamQ Universal SYBR qPCR Master Mix (Vazyme, China) was used for RT-PCR analysis. The relative mRNA expressions were determined using the ΔΔCt method with glyceraldehyde-3-phosphate dehydrogenase (GAPDH) serving as the endogenous control. In Table 1, specific primer sequences are listed. Every reaction was carried out three times to ensure reproducibility.

### 2.10. Western blotting

Protease inhibitor (Solarbio, China) and RIPA buffer (Beyotime.P0013B) were used to extract tissue protein samples. Protein samples were placed onto polyvinylidene fluoride membranes (Invitrogen, USA) after being separated on a 10% SDS-PAGE gel. The membranes were blocked for 1 h with 10% nonfat milk, and then primary antibodies against Papss2 (1:500, sc -100,801, Santa Cruz), and β-actin (1:1000, Cat#mAb3700, CST) were added for 12 h at 4°C, followed by HRP-linked anti-mouse IgG (1:5000, Cat#7076, CST) for 1 h. Using the ImageJ software, the intensities of protein bands were measured and the values were adjusted to β-actin.

### 2.11. Histologic analysis and immunostaining

Colon tissue was first fixed for one night using either 4% paraformaldehyde or Carnoy’s solution (30% chloroform, 60% methanol, 10% glacial acetic acid). It was then embedded in paraffin and cut into 5-μm-thick sections for staining with periodic acid Schiff (AB-PAS), H&E, and high iron diamine-alcian blue (HID-AB). In brief, for H&E staining, paraffin sections underwent a 2-min hematoxylin solution immersion followed by a quick dip in eosin solution for 10 repetitions and were observed under an optical microscope. On the basis of earlier research, the degree of histological alterations was graded. Paraffin sections were stained with an AB-PAS stain kit (Solarbio, China) for AB-PAS staining, the thickness of mucous layer was measured by Image J. For HID-AB staining, paraffin sections were stained with an HID-AB stain kit (Solarbio, China) and the sulfated acidic mucins were stained in shades of purple-brown to brown-black. Quantification of sulfomucin area was performed using Image J.

To perform immunostaining analysis on colon tissue, 0.3% (m/V) bovine serum albumin was used to block the sections for 1 h at room temperature. For immunofluorescence, the sections were treated at 4°C overnight with the primary antibody (anti-ZO-1 1:1000, Cat#A0659, ABclonal), then for 1 h with the corresponding secondary antibody. After PBS washing, the slices were mounted using 4’,6-diamidino-2-phenylindole (DAPI, C1002, Beyotime). A fluorescent microscope was used to examine and take pictures (Lycra, Germany). The slices were treated with the following primary antibodies respectively for immunohistochemistry: anti-MUC2 (1:1000, # 88686S, CST), anti-Papss2 (1:500, sc -100,801, Santa Cruz) at 4°C overnight. The slices were exposed to HRP-conjugated secondary antibodies for an hour the following day and signals were detected using diaminobenzidin (ZLI-9017, ZSGBBIO). A fluorescent microscope (Lycra, Germany) was used to check out and take pictures.

### 2.12. AHR nuclear translocation

On 12-well tissue culture chamber slides coated with poly-D-lysine (Sarstedt), HT29 cells (5 × 10^6^ cells/well) were cultivated overnight. Then, the cells were exposed to different compounds: vehicle (DMSO), IAA (100 μM), LPS (100 ng/ml), or IAA (100 μM) plus LPS (100 ng/ml) for 24 h. After treatment, following PBS rinsing, 4% (V/V) formaldehyde fixation, 0.3% (V/V) Triton X-100 permeabilization, and 3% (m/V) bovine serum albumin blocking, the cells were set aside. Subsequently, the cells were treated with the primary antibody against AHR (1:1000, ab308215, Abcam) at 4°C for 12 h and then followed by incubation with Alexa Fluor 594 labeled secondary antibody (ab150080, Abcam) for 1 h the next day. The nuclei were stained for 5 min with DAPI. Additionally, the negative control group was established by incubation with Alexa Fluor 594 labeled secondary antibody and performing DAPI staining without the primary antibody against AHR incubation. A fluorescent microscope (Lycra, Germany) was used to check out and take pictures.

### 2.13. High-throughput CUT&tag

CUT&tag assay was carried out according to previous directions^22^ by Shanghai Jiayin Biotechnology Ltd. (Shanghai, China). Briefly, after being extracted from frozen colon samples, about 500,000 native nuclei were incubated on magnetic beads coated with concanavalin A. After removing the unbound supernatant, the bead-bound cells were resuspended and treated with a primary antibody (AHR antibody 83,200, CST) or an IgG control antibody (normal rabbit IgG, Sigma Aldrich 12–370) overnight. After exposure to the secondary antibody (Goat anti Rabbit IgG H&L: abcam，ab6721) for 1 h the following day, the cells were rinsed and subjected to the pA-Tn5 adapter complex for 1 h, followed by suspension in tagmentation buffer (10 mM MgCl2 in Dig-med Buffer) and incubation for another 1 h. Next, DNA was purified and amplified to prepare libraries for sequencing. Finally, as directed by the manufacturer, 150 bp paired-end sequencing was carried out in the Illumina Novaseq 6000.

### 2.14. Statistical analysis

GraphPad Prism software was used for statistical analyses and visual representations. Data normality was assessed using Shapiro-Wilk test. The dataset was analyzed by one-way analysis of variance (ANOVA) followed by the post-hoc tests Tukey’s multiple comparisons test or Dunnett’s T3 multiple comparisons test for multiple group comparisons, or Student’s t-test for comparisons between two groups. Non-parametric test was employed to analyzed data that exhibited a skewed distribution. The collected data was displayed as mean ± standard deviation (SD), with p-value <0.05 denoting statistical significance (*p < 0.05, **p < 0.01, ***p < 0.001).

## 3. Results

### 3.1. Dietary fat exacerbated DSS-induced colitis

**Figure 1.**
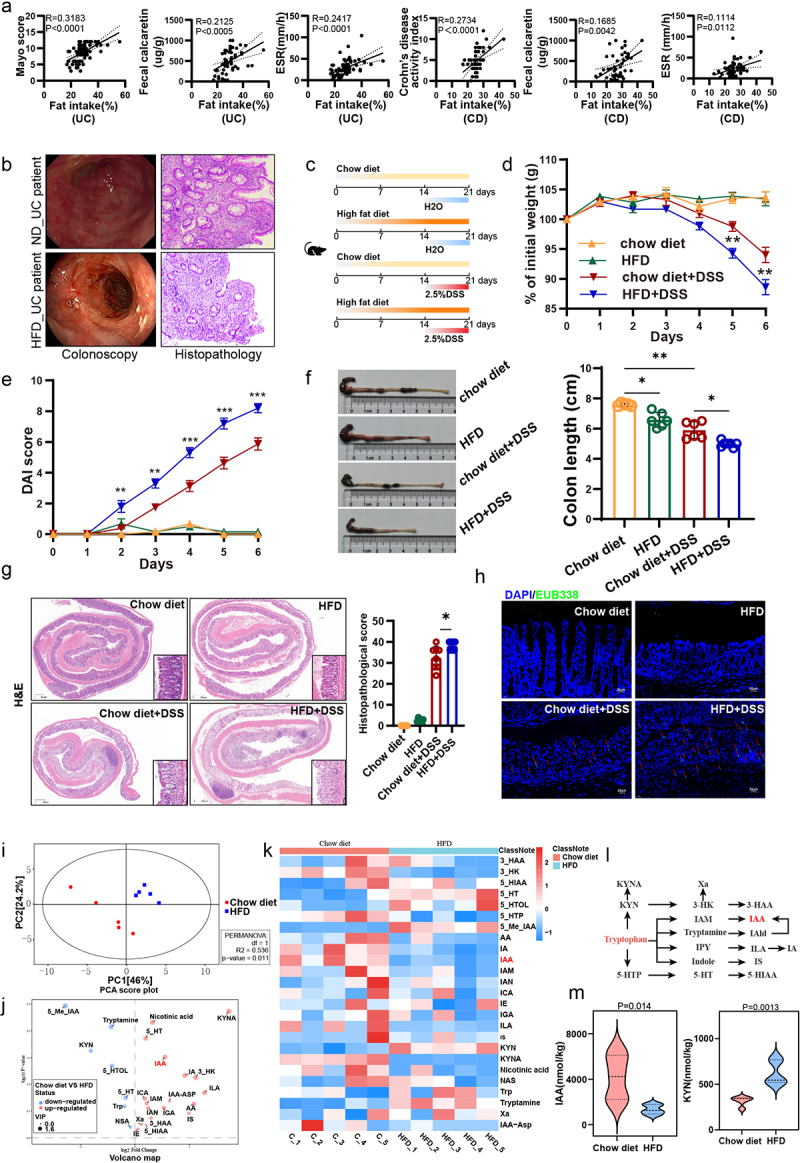
HFD exacerbated colitis in mice and induced a disruption of tryptophan metabolism in the gut.

To reveal the effects of HFD on intestinal homeostasis in patients with UC or CD, we conducted a retrospective dietary survey on 87 UC patients and 50 CD patients. Our findings revealed a positive correlation between dietary fat intake and disease activity indicators in both UC and CD patients, as shown by the Mayo score (*R* = 0.3183, *p* < 0.0001), fecal calprotectin levels (*R* = 0.2417, *p* < 0.0005) and ESR (*R* = 0.2417, *p* < 0.0001) in UC patients, Crohn’s disease activity index (*R* = 0.2734, *p* < 0.0001), fecal calprotectin levels (*R* = 0.1685, *p* = 0.0042) and ESR (*R* = 0.1114, *p* = 0.0112) in CD patients (Figure 1a). Furthermore, images of colonoscopy and histopathology suggested that UC patients on a HFD may develop more severe inflammation and mucosal damage (Figure 1b). To examine the effect of dietary fat on colitis, we first fed mice with either HFD or chow diet for 2 weeks and then administered them with 2.5% DSS before the significant difference in the mean body weight of HFD-fed mice and chow diet-fed mice appeared (Figure 1c). At harvest, mice that were fed a HFD had more DSS-induced body weight loss than chow diet-fed mice (Figure 1d). Moreover, the DAI score revealed HFD-fed mice had more sensitivity to DSS treatment and suffered from more serious aggressive colitis (Figure 1e). The colons of HFD-fed mice were shorter than controls and exhibited smaller cecum and obvious bloody erosion (Figure 1f). The histopathology revealed typical features of colitis with the reduced epithelial crypt density or increased crypt interspace, along with widespread infiltration of inflammatory cells and the presence of ulcers in DSS administration mice. A HFD exacerbated these phenomena, leading to an increased infiltration of inflammatory cells and the disappearance of crypts and an increased histopathological score compared to a chow diet (Figure 1g). Meanwhile, a HFD increased intestinal epithelial permeability, leading to increased bacterial translocation (Figure 1h). Collectively, these findings indicate that a short-term HFD exacerbates DSS-induced colitis and leads to intestinal barrier damage.

(a) Correlation analysis between fat intake and Mayo score, fecal calcaretin, and ESR in UC patients, as well as between Crohn’s disease activity index, fecal calcaretin, and ESR in CD patients. (b) Colonoscopic images (left panel) and colonic histopathological images (right panel) of UC patients with HFD and conventional diet. (c) Animal treatment procedure. C57BL/6 mice (*n* = 6) fed chow diet or HFD for 3 weeks and treated with H_2_O or 2.5% DSS in the last week. (d) Body weight changes. (e) Daily monitoring of DAI score including body weight loss, stool consistency, and fecal bleeding. (f) Representative images of the colon (left panel) and quantification of colon length (right panel). (g) Images of colon coil sections stained with H&E (left panel) and quantification of colon histopathological score (right panel). *Scale bars: 50 µm*. (h) Bacteria were detected by fluorescence in situ hybridization (FISH) using EUB338 probe. *Scale bars: 20 µm*. (i) PCA score plot from the mice with chow diet and HFD. (j) Volcano map from the mice with chow diet and HFD. (k) Heat map showed different content of tryptophan metabolites in chow diet mice and HFD mice. (l) Tryptophan metabolic pathway. (m) Changes of the levels of IAA and KYN in chow diet and HFD mice. *p < .05, **p < 0.01, ***p < .001.

### 3.2. Gut metabolome revealed a dramatic reduction of IAA in HFD-fed mice

Some studies have demonstrated that a HFD can alter the composition of gut microbiota, subsequently altering the microbial metabolic profiles.^23^ To delve deeper into the relationship between HFD and tryptophan metabolism, the targeted tryptophan metabolomics analysis of fecal samples from chow-diet and HFD-fed mice after 2 weeks of feeding was conducted. As a result, a partial but significant separation was observed between mice with chow diet-fed mice and HFD-fed mice as shown by principal components analysis (PCA) (Figure 1i). 7 and 18 modules were significantly enriched or depleted in HFD-fed mice, respectively (Figure 1j). Among these, metabolites from the kynurenine (kyn) and serotonin pathways were increased in the feces of HFD mice, such as kyn, with the exception of kynurenic acid (KYNA), which has been demonstrated to mitigate the severity of colitis by enhancing the proliferation of intestinal epithelial cells.^24^ Unfortunately, the production of indoles from indole pathway, which are crucial to intestinal homeostasis, is generally reduced in the feces of mice with HFD, including IAA and indole acrylic acid (IA) (Figure 1k-m).

### 3.3. IAA attenuated the severity of colitis and strengthened the gut barrier function

**Figure 2.**
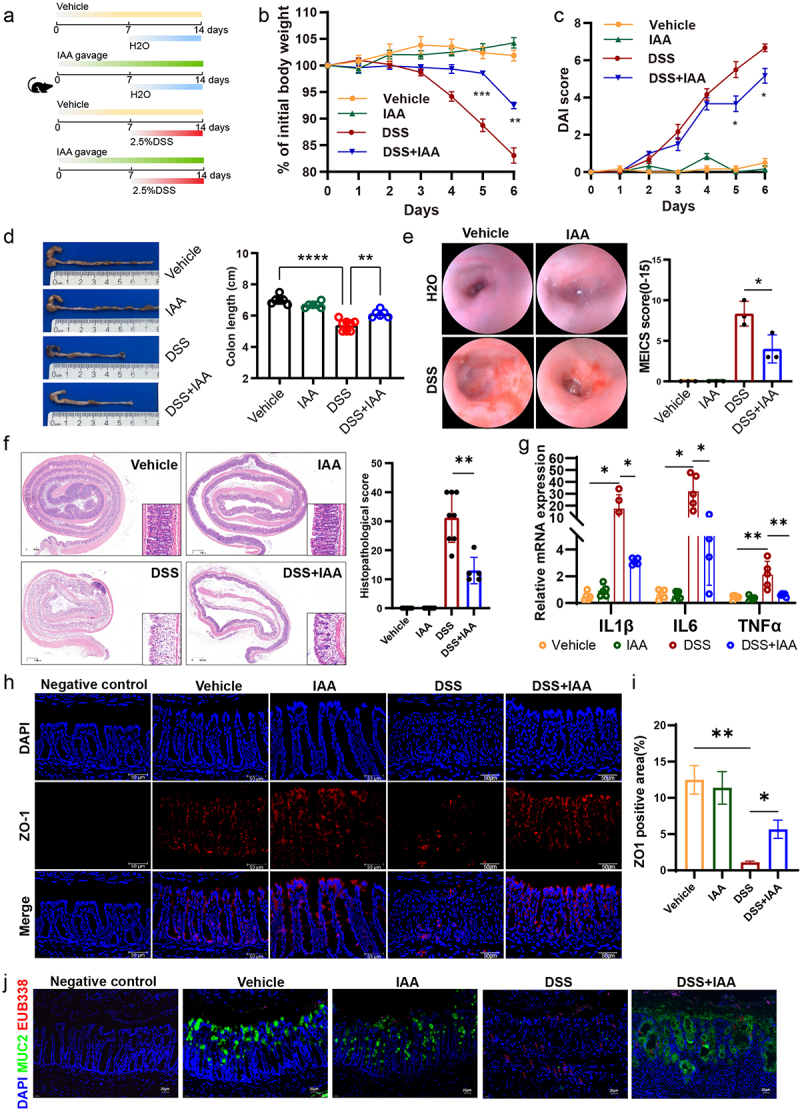
IAA ameliorated the severity of DSS-induced colitis and strengthened the gut barrier function.

The association between IAA and colitis remains relatively unexplored. To elucidate the potential role of IAA in colitis, it was administered orally into DSS-induced acute colitis mice 7 d in advance until the end of the experiment (Figure 2a). We found that DSS administration successfully resulted in obvious body weight loss on mice compared to the vehicle group receiving water. Nonetheless, administration of IAA to mice resulted in a significant mitigation of the weight loss induced by DSS (Figure 2b). To assess the severity of colitis, we monitored the DAI score daily during the modeling period. As a result, DSS markedly increased DAI score and IAA supplementation based on the DSS substantially reduced the DAI score, suggesting that IAA can alleviate the severity of colitis (Figure 2c). Meanwhile, prior to euthanizing the mice, the endoscopic examination showed DSS administration led to extensive mucosal erosion and bleeding within the intestinal lumen, with mucosal detachment observed in some cases, while IAA reduced the ulcer area and the extent of bleeding (Figure 2e). Consistently, IAA also significantly alleviated the reduction in colon length observed in mice challenged with DSS (Figure 2d). What’s more, in comparison to administering DSS alone, supplementation with IAA increased crypt number and crypt height, reduced infiltration of inflammatory cells, and mitigated epithelial cell damage and significantly decreased the pathological scores (Figure 2f). We further assessed the influence of IAA on inflammatory cytokines and found IAA feeding significantly decreased the levels of IL-1β, IL-6 and TNFα in colonic tissues of DSS-treated mice (Figure 2g). Immunofluorescence analysis revealed an increase in ZO-1 expression in the colitis model mice concomitantly treated with IAA compared with DSS treatment alone (Figure 2h-i). Additionally, DSS-treated mice showed increased bacterial infiltration into the epithelium, whereas IAA reduced bacterial translocation to the intestinal epithelium (Figure 2j).

(a) Animal treatment procedure. C57BL/6 mice (*n=*5-8) treated with or without IAA for 2 weeks and received H_2_O or 2.5% DSS in the last week. (b) Body weight changes. (c) Daily monitoring of DAI score including body weight loss, stool consistency, and fecal bleeding. (d) Representative images of the colon (left panel) and quantification of colon length (right panel). (e) Typical images of colonoscopy (left panel) and MEICS score (right panel). (f) Images of colon coil sections stained with H&E (left panel) and quantification of colon histopathological score (right panel) in DSS-induced colitis models. *Scale bars*: 300 *µ*m. (g) The relative mRNA expression levels of IL-1β, IL-6 and TNFα. (h) Immunofluorescence staining of ZO-1 protein. *Scale bars*: 50 *µ*m. (i) ZO-1 positive area statistics. (j) FISH using EUB338 probe for bacterial 16S ribosomal RNA (pink) as well as immunofluorescence of MUC2 (green) in colon tissue. Scale bars: 20 µm. *p < .05, **p < .01, ***p < .001.

To further clarify the effect of IAA, we used TNBS to induce colitis and observed similar findings. IAA administration decreased the mouse mortality, weight loss, colonic shortening, and mitigated mucosal epithelial damage induced by TNBS (Figure S2a-d). Furthermore, IAA-treated colitis mice exhibited lower levels of pro-inflammatory cytokines including IL-1β and TNFα (Figure S2e) than the colitis mice.

To better evaluate the therapeutic potential of IAA in colitis and to mitigate any potential interference of IAA with the pharmacological effects of DSS, we initially administered mice with drinking water containing DSS for 7 d. Subsequently, upon the onset of noticeable colitis symptoms, the mice were treated by IAA on the 8th day for 5 d (Figure S3a). The results showed that mice in the IAA group had faster recovery of intestinal damage, lower intestinal permeability and reduced inflammation compared to the control group (Figure S3b-k).

Collectively, these data demonstrated that IAA treatment could alleviate colonic inflammation and restore epithelial damage in colitis model mice.

### 3.4. IAA enhanced the mucosal barrier function by promoting mucin sulfation

**Figure 3.**
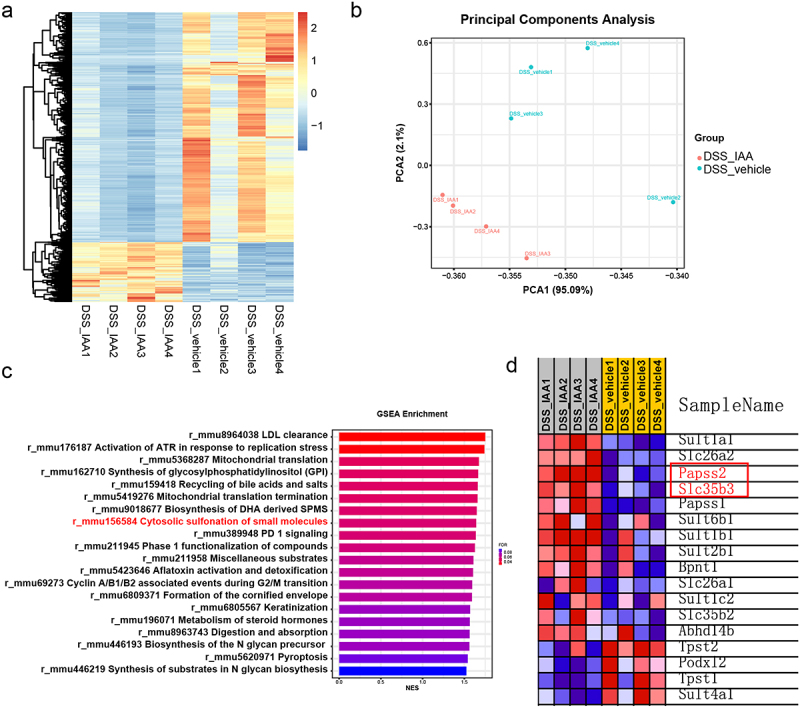
Transcriptomic analysis revealed IAA promoted the enrichment of the sulfation pathway.

**Figure 4.**
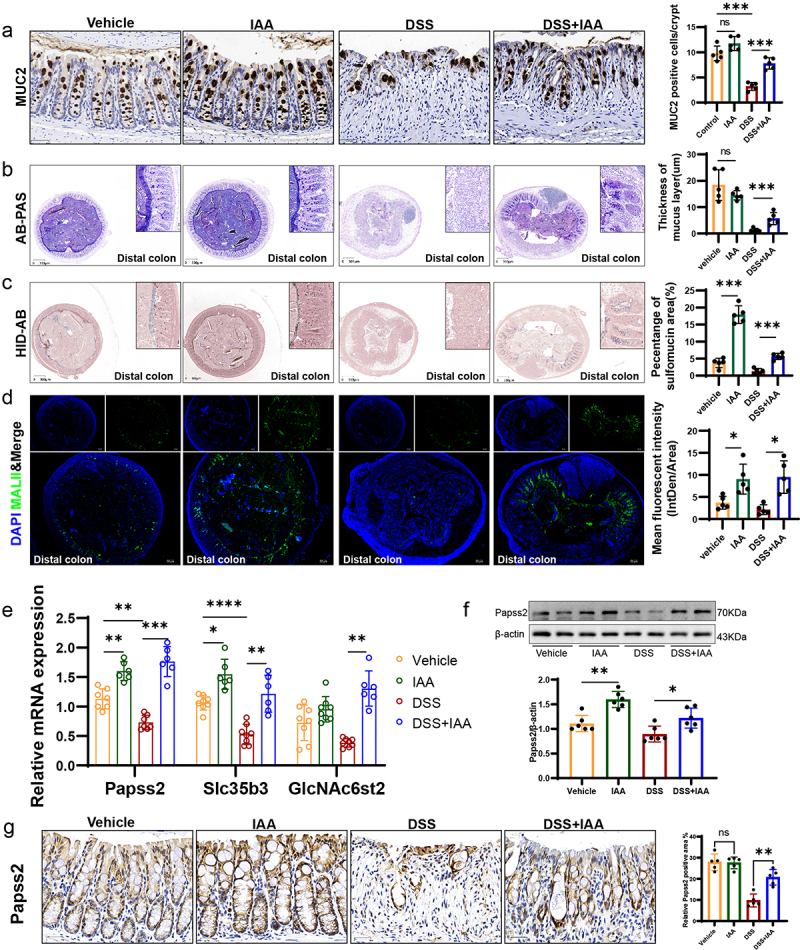
IAA supplementation enhanced the mucosal barrier function by promoting mucin sulfation.

In the in vivo experiments, we further observed a notable increase in goblet cells number and mucous layer thickness in mice supplemented with IAA compared to the DSS-treated group (Figure 4a-b, Figure S1a-b). To further mine the mechanisms underlying the improvements of colonic mucus layer in IAA-treated mice, we performed the RNA sequencing analysis of the colonic tissues from DSS-treated mice with or without IAA. The results illustrated significant differences in the transcriptomes between two groups (Figure 3a-b). The addition of IAA to DSS treated mice repressed the gene expression of inflammatory cytokines such as IL-6, IL-1β, and Cxcl2 (Figure S4b). Furthermore, the analysis of differentially expressed genes using the Kyoto Encyclopedia of Genes and Genomes (KEGG) pathway revealed a significant enrichment in multiple signaling pathways. Among them, the sulfur metabolism signaling was significantly enriched (Figure S4a). The gene set enrichment analysis (GSEA) showed IAA treatment altered the cytosolic sulfonation pathway (Figure 3c,d). The process of sulfation, facilitated by sulfotransferases, is a crucial conjugation reaction, and sulfonated carbohydrates constitute a significant portion of mucin composition.^25^ Sulfation frequently takes the form of terminal caps in mucins, which normally prevent oligosaccharide enzymatic breakdown.^26^ 3'-phosphoadenosine 5'-phosphosulfate (PAPS) is the sulfate donor and Papss2 is the key enzyme to catalyze the formation of PAPS. Then, PAPS translocases Slc35b3 are responsible for transporting PAPS to Golgi apparatus to participate in the mucin sulfation reaction.^27–29^ Intestinal deficiency of Papss2 has been reported to compromise mucin sulfation and gut barrier on colitis challenge.^30^ In our study, the RNA sequencing data showed a significant increase in Papss2 and Slc35b3 expression in IAA-treated colitis mice (Figure 3d). Additionally, we conducted RNA sequencing analysis on mice supplemented with or without IAA but without DSS and a significant difference in the transcriptomes between these two groups was observed (Figure S4c). However, we did not observe enrichment of the cytosolic sulfonation pathway. Nonetheless, we did find increased mRNA expression of Papss2 in the IAA group (Figure S4d). The results were confirmed by RT-PCR (Figure 4e).

(a) Heatmap of the differentially expressed genes according to the value of |logFC| in DSS/DSS+IAA mice. (b) PCA score plot from DSS/DSS+IAA mice. (c) GSEA of genes in the colon tissues of DSS/DSS+IAA mice. (d) Heatmap of genes expression of the cytosolic sulfonation pathway in DSS/DSS+IAA mice.

(a) Representative micrographs of MUC2 immunohistochemistry (left panel) and quantification of MUC2 positive cells/crypt (right panel) in colon sections from different groups of mice. *Scale bars*: 30 *µ*m. (b) Representative micrographs (left panel) and quantification (right panel) of AB-PAS staining (mucus layer was stained blue or purple) in distal colon sections from different groups of mice. *Scale bars*: 300 *µ*m. (c) Representative micrographs (left panel) and quantification (right panel) of HID-AB staining (sulfomucin was stained brown) in distal colon sections from different groups of mice. *Scale bars*: 300 *µ*m. (d) Immunofluorescence staining of DAPI (blue) and MALII lectin (green) in the distal colons (left panel) and quantification of the mean fluorescent intensity (MFI) (right panel). *Scale bars: 50 µm*. (e) The relative mRNA expression levels of Papss2, Slc35b3, and GlcNAc6st2 in the colon of mice by quantitative RT-PCR. (f) The protein expression of Papss2 in the colon of different groups of mice by western blot and quantification of the value of Papss2/β-actin. (g) Representative micrographs of immunohistochemical detection of Papss2 in mice colonic tissues (left panel) and quantification of the abundance of Papss2 (right panel). *Scale bars*: 30 *µ*m. *ns*, not significant, *p < .05, **p < .01, ***p < .001.

HID-AB staining and the lectin MALII staining allow for specific visualization of the sulfomucin subtype of mucins.^31,32^ In our study, we observed that the sulfated mucin was markedly reduced in the colonic tissues in DSS-treated mice compared with untreated mice, while IAA increased the sulfated mucin content in the epithelium of colon, especially in the distal colon (Figure 4c,d; Figure S1c-e). Moreover, we observed a reduction in the expression of Papss2 and Slc35b3 in the DSS group compared to the control groups and supplementation of IAA on the basis of DSS administration restored the expression of them, as we previously mentioned (Figure 4e). Consistently, IAA treatment restored the protein expression of Papss2 in the DSS-treated colonic tissues (Figure 4f-g). We also found that IAA promoted the expression of GlcNAc6st2 (Figure 4e), which has been reported to be the major sulfotransferase in the sulfation modification of mucins in mouse colon.^33^

**Figure 5.**
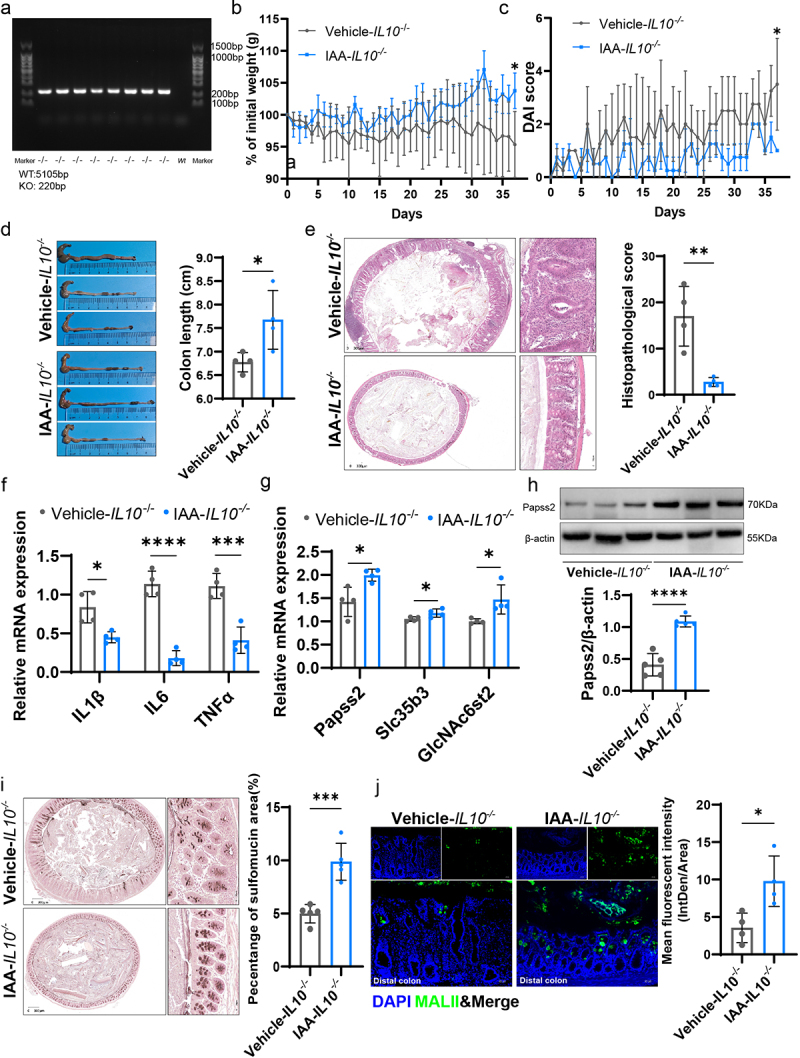
IAA supplementation ameliorated the spontaneous colitis and promoted mucin sulfation in *IL10*^*-/-*^ mice.

On the basis of identifying and verifying the genotype of *IL-10*^*-/-*^ mice (Figure 5a). We supplemented 11-week-old *IL-10^−/−^* mice with or without IAA and observed that by the time the mice reached 16 weeks, the vehicle group developed significant spontaneous colitis, whereas the IAA group did not exhibit noticeable inflammation. Specifically, compared to the vehicle group, the IAA group had a lower DAI score, less weight loss, less colonic shortening, lower histopathological scores, and lower levels of inflammatory cytokines (Figure 5b-f). Additionally, using HID-AB staining and MALII lectin staining to assess the levels of sulfated mucins in both groups, we found that the IAA group had higher levels of sulfated mucins in the proximal, middle or distal colon (Figure 5i,j; Figure S5a-d). Moreover, IAA supplementation promoted the mRNA expression of Papss2, Slc35b3, and GlcNAc6st2 (Figure 5g), as well as the protein expression of Papss2 (Figure 5h).

(a)The mouse genotypes were determined through PCR to detect wild-type and mutant alleles of *IL10*, utilizing designated primers. (b) Body weight changes. (c) Daily monitoring of DAI score including body weight loss, stool consistency, and fecal bleeding. (d) Representative images of the colon (left panel) and quantification of colon length (right panel). (e) Images of colon tissues stained with H&E (left panel) and quantification of colon histopathological score (right panel) in a spontaneous model of colitis. *Scale bars: 300 µm*. (f) The relative mRNA expression levels of IL-1β, IL-6, and TNFα. (g) The relative mRNA expression levels of Papss2, Slc35b3, and GlcNAc6st2 by quantitative RT-PCR. (h) Protein expression of Papss2 in *IL10*^*-/-*^ mice with or without IAA by western blot and quantification of the value of Papss2/β-actin. (i) Representative micrographs of HID-AB staining (sulfomucin was stained brown) from the distal colon sections in *IL10*^*-/-*^ mice with or without IAA (left panel) and quantification of the percentage of sulfomucin area (right panel). *Scale bars: 300 µm*. (j) Immunofluorescence staining of DAPI (blue) and MALII lectin (green) (left panel) and quantification of the mean fluorescent intensity (MFI) (right panel) from the distal colons in *IL10*^*-/-*^ mice with or without IAA. Scale bars: 50 µm. *p < .05, ***p < .001, ****p < .0001.

**Figure 6.**
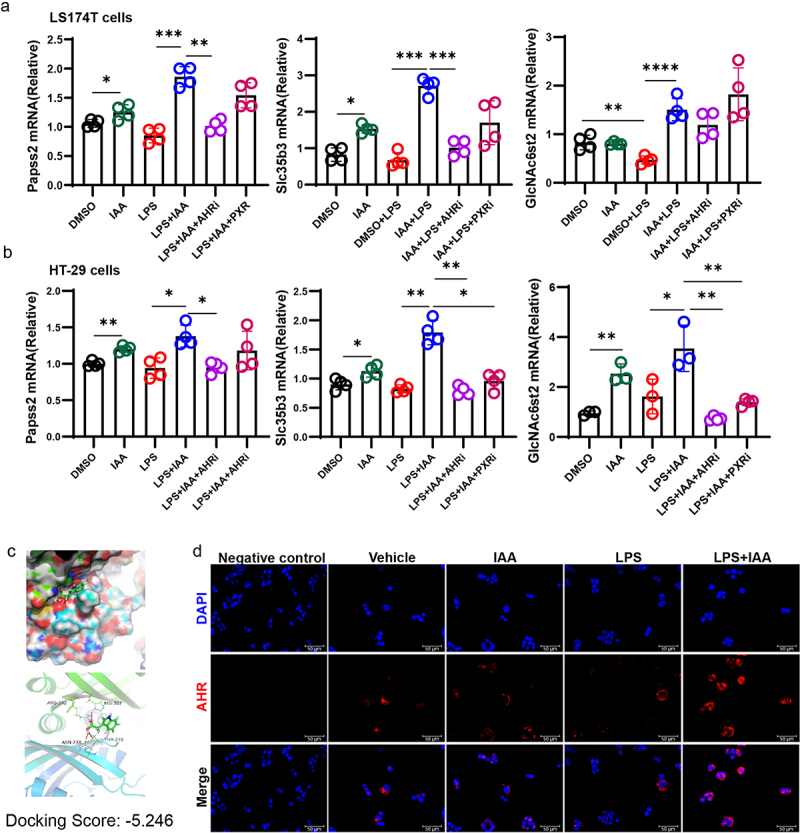
IAA activated AHR to regulated mucin sulfation.

Furthermore, we investigated the effect of IAA on mucin sulfation in LS174T and HT29 cells in vitro and found that combined application of IAA and LPS led to higher mRNA expression levels of Papss2 and its downstream genes including Slc35b3 and GlcNAc6st2 than LPS alone (Figure 6a-b). These results indicated that IAA improved mucus barrier function by partially strengthening sulfation of mucin.

(a) The relative mRNA expression levels of Papss2, Slc35b3, and GlcNAc6st2 in LS174T cells by quantitative RT-PCR. (b) The relative mRNA expression levels of Papss2, Slc35b3, and GlcNAc6st2 in HT-29 cells by quantitative RT-PCR. (c) Molecular docking analysis of IAA binding on the ligand-binding domain of AHR protein. The binding energy is −5.246 kcal/mol. (d) Immunostaining images of HT-29 cells stained for DAPI (blue), AHR (red), and merge. *Scale bars*: 50 *µ*m. *p < .05, **p < .01, ***p < .001, ****p < .0001.

### 3.5. Effects of IAA on mucin sulfation were partially dependent on AHR

Tryptophan in the colon can be metabolized by the gut microbiota into a range of indole metabolites including IAA.^10^ It has been reported that IAA functions as the AHR or PXR ligand.^34,35^ To verify which receptor plays a role in IAA-mediated increase in Papss2 expression, LS174T and HT29 cells were pretreated with PXR and AHR inhibitors in vitro, prior to LPS and IAA treatments. However, we found that only the AHR inhibitor significantly suppressed Papss2 expression, with no statistically significant differences were observed between the LPS+IAA and LPS+IAA+PXRi groups (Figure 6a,b). Consequently, we conclude that AHR plays a predominant role in inducing Papss2 expression. Molecular docking analysis revealed that IAA bound to the ligand-binding domain of AHR with a binding energy of −5.246 kcal/mol, which indicates that IAA exhibits a notable capacity for binding to AHR (Figure 6c). In addition, we observed increased AHR nuclear translocation after the cells were exposed to LPS and IAA together (Figure 6d). These data suggested that IAA could bind to AHR to promote the entry of AHR complex into the nucleus and then regulate the expression of target genes.

**Figure 7.**
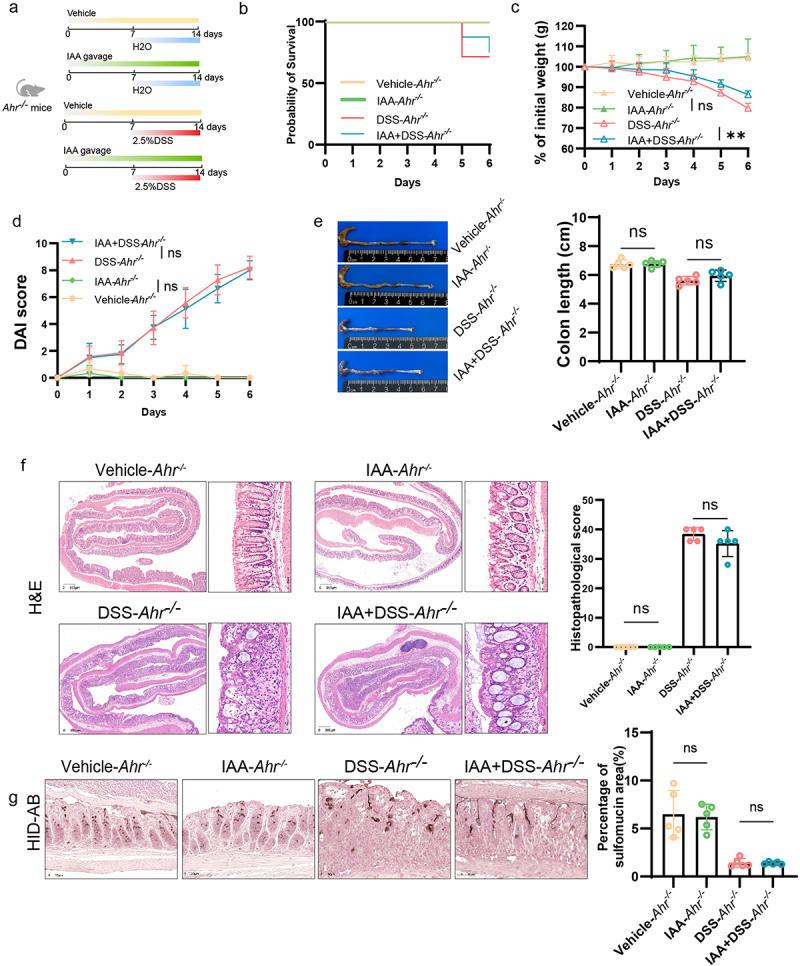
Effects of IAA on mucin sulfation were partially dependent on AHR.

**Figure 8.**
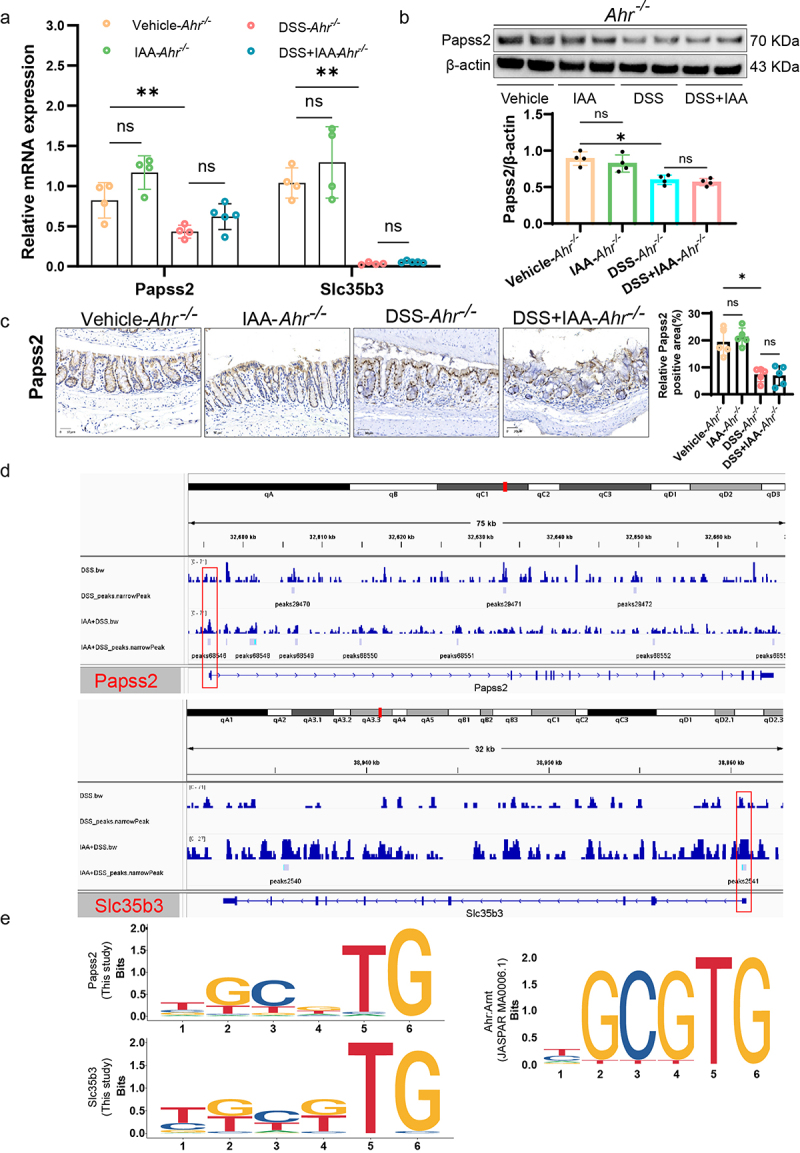
AHR-XRE interaction mediated the effect of IAA on Papss2.

To further investigate whether the impacts of IAA on intestinal mucin sulfation are orchestrated through the mediation of AHR, we administered IAA to *Ahr^−/−^* mice, followed by DSS challenge (Figure 7a). The results indicated that in *Ahr^−/−^* mice, IAA alleviated DSS-induced weight loss but did not improve survival rate, DAI score, colon shortening, or histopathological score (Figure 7b-e). We concluded that supplementation of IAA in *Ahr^−/−^* mice did not ameliorate the damage to intestinal epithelium caused by DSS (Figure 7f). The HID-AB staining unveiled that AHR knockout counteracted the elevated sulfomucin levels induced by IAA (Figure 7g). Critically, our investigation encompassed quantitative RT-PCR of intestinal tissue, revealing that IAA administration reversed the diminution of Papss2 and Slc35b3 levels evoked in vivo by DSS, such effects were attenuated in *Ahr^−/−^* mice (Figure 8a). Consistently, the protein expression level of Papss2 showed similar results (Figure 8b,c).

(a) Animal treatment procedure. *Ahr*^*−/−*^ mice (*n* = 5) treated with or without IAA for 2 weeks and received H_2_O or 2.5% DSS in the last week. (b) Survival curve of *Ahr*^*−/−*^ mice. (c) Body weight changes in *Ahr*^*−/−*^ mice. (d) Daily monitoring of DAI score in *Ahr*^*−/−*^ mice, including body weight loss, stool consistency, and fecal bleeding. (e) Representative images of the colon (left panel) and quantification of colon length (right panel) from *Ahr*^*−/−*^ mice. (f) Histological images of H&E-stained colonic tissues (left panel) and colon histopathological score (right panel). *Scale bars*: 300 *µ*m. (g) Representative micrographs (left panel) and quantification (right panel) of HID-AB staining (sulfomucin stains brown) in colon sections from *Ahr*^*−/−*^ mice with or without IAA for 2 weeks and received H_2_O or 2.5% DSS for 7 d. *Scale bars*: 30 *µ*m. ns, not significant, **p < .01.

(a) The relative mRNA expression levels of Papss2, Slc35b3 in *Ahr*^*−/−*^ mice by quantitative RT-PCR. (b) Protein expression of Papss2 in *Ahr*^*−/−*^ mice by western blot (left panel) and quantification of the value of Papss2/β-actin (right panel). (c) Representative micrographs of immunohistochemical detection of Papss2 (left panel) and quantification of the expression of Papss2 (right panel) in colon tissues of *Ahr*^*−/−*^ mice. *Scale bars*: 30 *µ*m. (d) CUT&tag assay showed AHR binds to the promoter region of Papss2 and Slc35b3. (e) Logo representation of the motif. ns, not significant, *p < .05, **p < .01.

### 3.6. IAA increased Papss2 expression via AHR-XRE interaction in the Papss2 promoter

As a transcription factor, AHR is transferred to the nucleus after binding ligands, and then binds to DNA to regulate the transcription of target genes.^13^ In both in vivo and in vitro experiments, we found that AHR inhibitors or AHR knockout partially abolished the promotive effect of IAA on Papss2 gene expression. Therefore, we hypothesize that Papss2 may be a target gene of AHR. Further employing Cut&tag experiments, we found an enrichment of AHR in the gene promoter regions of Papss2 as well as Slc35b3. This suggested that AHR could directly regulate the transcription of Papss2 and Slc35b3 genes (Figure 8d). We further analyzed the binding sites of AHR on the Papss2 and Slc35b3 promoters and found the presence of XRE (TGCGTG/CGCGTG) in both Papss2 and Slc35b3 promoter regions, consistent with the AHR binding motif in the JASPAR database (Figure 8e). These results indicated that AHR directly regulated the transcription of both genes by binding to XREs in the promoter regions of Papss2 and Slc35b3 and further regulated the sulfation process.

### 3.7. Lactobacillus reuteri-derived IAA was required and sufficient to alleviate colitis and promote sulfation of mucin

**Figure 9.**
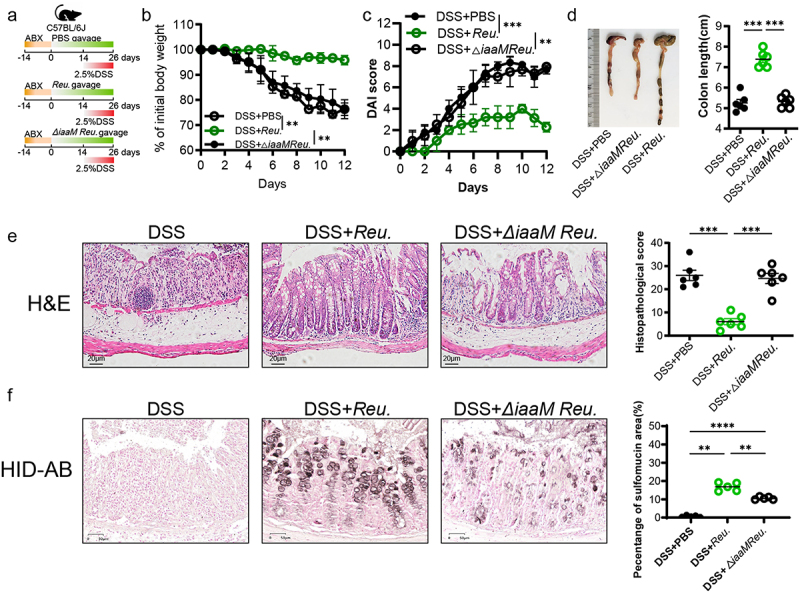
*L. reuteri*-derived IAA was required and sufficient to alleviate colitis and promote sulfation of mucin.

*L. reuteri* has been shown to produce several indole derivatives, including IAA and I3A.^35,36^ To further elucidate the primary role of IAA in ameliorating colitis, we engineered a mutant strain of *L. reuteri* lacking the *iaaM* gene (*Lactobacillus*^*ΔiaaM*^), resulting in the inability to produce IAA. We evaluated the IAA production capabilities of *L. reuteri* and *Lactobacillus*^*ΔiaaM*^ in vitro (Figure S6a). Subsequently, we administered oral gavage of PBS, *L. reuteri*, or *Lactobacillus*^*ΔiaaM*^ to C57BL/6J mice. These mice had their gut microbiota depleted with an antibiotic cocktail (ABX) administered 2 weeks prior (Figure 9a). After confirming the successful colonization of *L. reuteri* and *Lactobacillus*^*ΔiaaM*^ (Figure S6b), we initiated concurrent DSS treatment in the last 12d. The results showed that *L. reuteri* mitigated the increased weight loss, high DAI score and reduced colon length caused by DSS and there was a significant inflammation improvement in mice from *L. reuteri* gavage group compared with DSS treatment alone (Figure 9b-e). The *Lactobacillus*^*ΔiaaM*^ gavage group, however, did not differ substantially from that in the DSS group. HID-AB staining revealed a significant increasing in colonic sulfomucin content in goblet cells after oral administration of *L. reuteri* compared to the PBS group. However, compared to *L. reuteri*, *Lactobacillus*^*ΔiaaM*^ exhibited a significantly reduced capacity to promote mucin sulfation (Figure 9f). The above results suggest that IAA is essential in protecting *L. reuteri* against colitis and promoting mucin sulfation.

(a) Animal treatment procedure. C57BL/6 mice (*n* = 6) were administered an ABX for 2 weeks, then separately gavaged with PBS, *Reu*. and *△iaaM Reu*. for 26 d and received PBS or 2.5% DSS in the last 12 d. (b) Body weight changes. (c) Daily monitoring of DAI score. (d) Representative images of the colon (left panel) and quantification of colon length (right panel). (e) Histological images of H&E-stained colonic tissues (left panel) and colon histopathological score (right panel). *Scale bars*: 20 *µ*m. (f) Representative micrographs (left panel) and quantifications (right panel) of HID-AB staining (sulfomucin stains brown) in colon sections. *Scale bars*: 50 *µ*m. ns, not significant, *p < .05, **p < .01, ***p < .001, ****p < .0001. ABX, antibiotic cocktail; *△iaaM Reu*., *Lactobacillus^ΔiaaM^*; *Reu*., *L. reuteri*;.

### 3.8. Papss2 expression and mucin sulfation level were down-regulated in UC patients exposed to HFD

**Figure 10.**
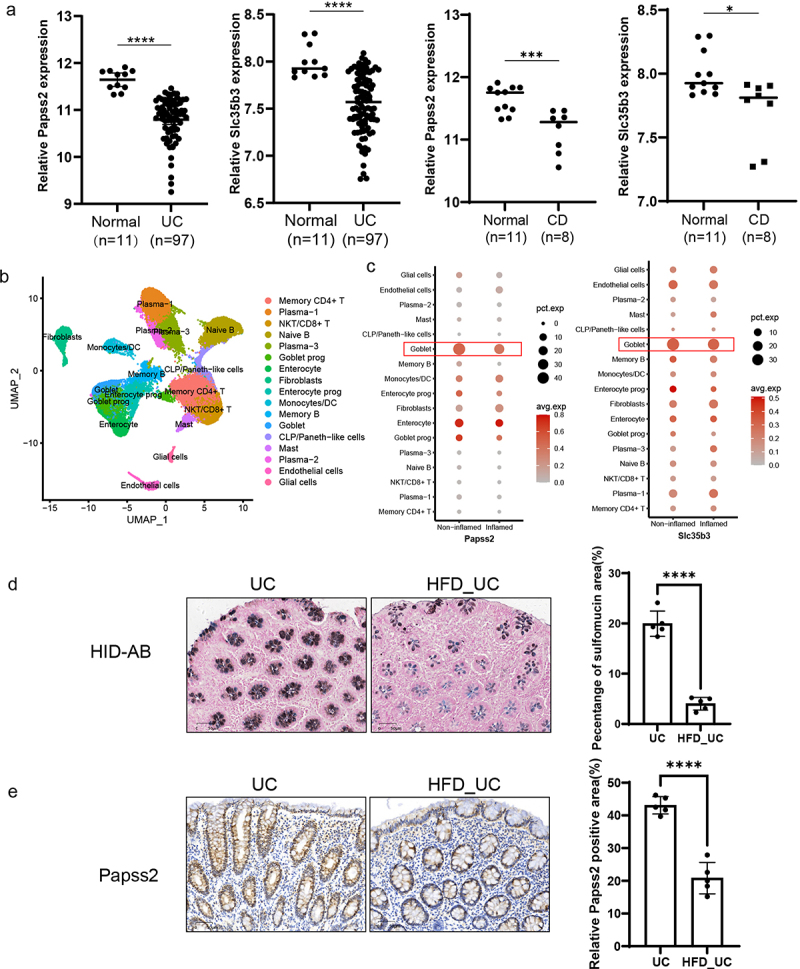
Expression of Papss2 and mucin sulfation were down-regulated in UC patients exposed to HFD.

Since the expression of Papss2 and the abundance of mucin sulfation exhibited reduced expression in murine colitis, exploring its expression profile in patients with IBD holds substantial significance. Analysis of transcriptomic data from colonic tissues in the Gene Expression Omnibus (GEO) database (GSE59071) indicated a notable reduction in the expression of Papss2 and Slc35b3 in both UC and CD patients compared to healthy individuals (Figure 10a). Single-Cell RNA sequencing from inflamed tissue of the descending colon and non-inflammatory tissue of the ascending colon in patients with left-sided UC revealed reduced expression of Papss2 and Slc35b3 in goblet cells within the inflamed tissues compared to non-inflamed tissues (Figure 10b,c), which were consistent with the GEO database, suggesting that the reduction in sulfation of goblet cell mucins may be closely associated with the occurrence of inflammation. In the preceding dietary survey, the association between a HFD and disease activity in IBD was identified. Finally, we conducted HID-AB staining and immunohistochemical staining for Papss2 on colon tissue sections from UC patients and those with UC exposed to HFD. The results showed the level of mucin sulfation and the expression of Papss2 in UC patients exposed to HFD were obviously lower than in UC patients (Figure 10d,e). In conclusion, the aforementioned results suggested a close association between the Papss2 and sulfation and IBD, as well as HFD.

(a) The gene expression of Papss2 and Slc35b3 in colonic mucosa from UC patients (*n* = 97), CD patients (*n* = 8) and normal individuals (*n* = 11) (GSE59071). (b) Single-cell RNA sequencing analysis reveals cell types annotated in colon tissue biopsy specimens. (c) The relative expression of Papss2 and Slc35b3 in goblet cells of inflammatory and non-inflammatory colon biopsy tissue samples from patients with UC. (d) Representative micrographs (left panel) and quantifications (right panel) of HID-AB staining (sulfomucin stains brown) in colon sections from UC patients with or without HFD diet. *Scale bars*: 50 *µ*m. (e) Representative micrographs of immunohistochemical detection of Papss2 in colon sections from UC patients with or without HFD diet (left panel) and quantifications of the abundance of Papss2 (right panel). *Scale bars*: 50 *µ*m. *p < .05, ***p < .001, ****p < .0001.

## 4. Discussion

Growing evidence suggests that western HFD affects the risk of IBD.^37,38^ In our study, we also demonstrated that a HFD could aggravate the severity of colitis and disrupt gut barrier function in DSS-induced colitis model. Previous studies have revealed that a HFD could shape the metabolism of active tissues, such as the immune cells^35,39^ and adipose cells.^40^ In an effort to preempt the potential influence of metabolic alterations induced by diet on intestinal inflammation, we exclusively administered a 2-week regimen of HFD to the mice. Prior to DSS-induced colitis modeling, comparative analysis with the chow diet revealed that the HFD did not elicit significant fluctuations in the mice's body weight, which excluded that HFD affected colitis by altering cellular metabolism.

A few studies have reported that HFD depleted levels of certain gut metabolites and then increased the host’s susceptibility to pathogenic elements.^41–43^ In our previous experiment, we found maternal HFD disrupted intestinal mucus barrier of offspring might by changing gut microbial tryptophan metabolism and reducing the levels of microbiota- associated indole metabolites. We further investigated the specific impact of a HFD on tryptophan metabolism and also found mice fed with HFD displayed a distinctive tryptophan metabolomic profile compared with chow diet-fed mice. Specifically, HFD increased level of Kyn and decreased level of IAA in the fecal samples. The result is supported by the findings reported in HFD-induced metabolic syndrome and nonalcoholic fatty liver disease mouse models, inconsistently, in which mice were fed with HFD 12 weeks and 8 weeks.^42,43^ Notably, higher Kyn level and lower IAA level were also observed in patients with IBD and subjects with obesity.^15^ A recent study discovered HFD could disrupt serum tryptophan metabolism and upregulate the kynurenine metabolic pathway,^44^ consistent with our findings. Some microbiota-derived tryptophan metabolites have been reported to play important roles in reducing colonic inflammation and protecting the integrity of intestinal epithelium.^9^ We therefore propose the hypothesis that HFD dysregulates intestinal tryptophan metabolism, leading to a decrease in IAA levels, which in turn deprives IAA of its protective effect against HFD-induced inflammation and disruption of intestinal barrier function.

Our subsequent in vivo experiment verified that IAA could significantly relieve colitis and protect the gut barrier integrity. This was further supported by the results of RNA sequencing analysis. When compared to control colitis animals, the expressions of inflammatory markers such as IL-1β, IL-4, IL-6, and Cxcl2 were significantly lower in mice receiving IAA treatment. Mechanistically, we observed the cytosolic sulfation pathway was enriched in DSS plus IAA group, in which the expressions of Papss2 and Slc35b3 were significantly elevated. Both of them are essential in the process of mucin sulfation modification. Within the colon, O-glycan chains are extensively modified by sulfation to impede enzymatic degradation of the glycan chains by bacteria. Bacteria encoding sulfatases can recognize sulfated glycans and desulfate them, subsequently breaking down mucin glycan chains to obtain a source of energy. Clinical investigations have indicated elevated sulfatase activity of fecal mucins in IBD.^45,46^ Correspondingly, the reduction in mucin sulfation levels may potentially foster the proliferation of mucus-degrading bacteria, such as *Bacteroides fragilis*,^26^ within the mucus layer, thereby promoting their colonization and growth. This heightened bacterial translocation could, in turn, trigger inflammatory responses in susceptible hosts. The function of the intestinal mucosal barrier is critically dependent on the sulfation of mucins. Mice without sodium sulfate cotransporter 1 (NaS1), the sulfate transporters, exhibited reduced mucin sulfation and had increased susceptibility to experimental colitis.^47^ The sulfation of mucins involves sulfotransferases transferring SO3^−^ from PAPS to the O-glycan chains of mucins. PAPS, a universal sulfation donor in vivo, is synthesized by Papss1 and Papss2. Conventionally, it has been proposed that Papss1 predominantly localizes to the nucleus across various cell types, while Papss2 primarily resides in the cytoplasm.^48^ Contrary to these notions, our immunohistochemical analysis revealed the presence of Papss2 within the cell nucleus in our experimental setting, necessitating further investigation into its specific functions. Mice deficient in Papss2 display reduced intestinal sulfomucin content and heightened sensitivity to DSS-induced colitis. Additionally, the diminished expression of Papss2 is correlated with poorer survival rates in colorectal cancer patients, indicating its potential significance beyond IBD. Furthermore, Papss2 defects have been linked to skeletal developmental abnormalities, highlighting the broader implications of identifying regulatory targets for Papss2 not only in the context of IBD but also in diseases associated with colorectal cancer and skeletal development.^30^ In addition, within mucins, SO3^−^ may attach to the 6-hydroxyl of N-acetyl-D-glucosamine (6S-GlcNAc) and terminal D-galactose (Gal) sugars at hydroxyl positions 3, 4, or 6 (3S-Gal, 4S-Gal, and 6S-Gal, respectively).^26,49^ GlcNAc6st2, in contrast to GlcNAc6st1, was reported to be the main sulfotransferase in the biosynthesis of colonic sulfated mucins in mice. The deficiency of GlcNAc6st2 greatly increased leukocyte infiltration in colon in DSS-induced colitis and butyrate can induce GlcNAc6st2 expression in colonic epithelial cells.^33^ In our work, we also examined the GlcNAc6st2 expression and found IAA could similarly enhance GlcNAc6st2 expression, possibly through direct or indirect mechanisms. These findings emphasize the complex regulatory pathways governing mucin sulfation, underscoring the potential interplay between environmental factors, like butyrate and IAA, in modulating GlcNAc6st2 expression in colonic epithelial cells.

IAA has traditionally been considered a product synthesized by *L. reuteri*.^15^ The enzyme iaaM, pivotal in the conversion of tryptophan to IAA, was engineered as a key rate-limiting step in this pathway.^36^ In our investigation, we generated a *L. reuteri* strain with an *iaaM* geen deficiency, and observed that the wild-type *L. reuteri* promoted mucin sulfation, while the *iaaM*-deficient strain (*Lactobacillus*^*ΔiaaM*^) exhibited a significantly reduced capacity to enhance mucin sulfation. Our findings underscore the critical role of IAA in modulating mucin sulfation. Notably, a longstanding observation has indicated diminished mucin sulfation in patients with UC,^25^ yet mechanistic insights have remained elusive. Therefore, the identification of pertinent targets to improve mucin sulfation is highly anticipated. Our research not only elucidates the link between tryptophan metabolites and mucin sulfation but also investigates the underlying mechanisms. Tryptophan-related indole metabolites have been reported for their potential to improve intestinal barrier function.^9^ Consequently, our subsequent efforts involve modifying IAA to enable targeted release in the colon, with the goal of enhancing its therapeutic efficacy in alleviating colitis. This will lay a more robust groundwork for the clinical implementation of IAA in managing IBD.

Collectively, these findings addressed the crucial role of colonic mucin sulfation in maintaining colon’s mucosal homeostasis and identified the potential therapeutic targets for IBD. However, few studies have illustrated the exact regulatory mechanisms for these targets. In the present study, we revealed that IAA could upregulate the intestinal expression of Papss2 through AHR. Meanwhile, AHR can bind to the promoter of Papss2 and initiate its transcription. The upregulation of Papss2 expression increased the biosynthesis of intracellular PAPS and the upregulation of Slc35b3 facilitated the translocation of PAPS into golgi apparatus, together these promoted the sulfation of mucins. Our research adds to the growing body of data suggesting that changes in the biochemical components of the mucus may lead to IBD pathogenesis, specifically mucin glycan sulfation modification. This offers another treatment perspective based on the present anti-inflammatory or other immune-based treatments.

## Supplementary Material

Supplemental Material

SemiQuanttative Food Frequency questionnaire.docx

## Data Availability

The data underlying this article have been deposited to the NCBI’s GEO. The RNA-seq data are available at: https://www.ncbi.nlm.nih.gov/geo/query/acc.cgi?acc=GSE249996. The CUT&Tag data are available at: https://www.ncbi.nlm.nih.gov/geo/query/acc.cgi?acc=GSE255130 (Secure token is “ybkjmqmufpqbpcd”). The single cell RNA-seq data are available at: https://www.ncbi.nlm.nih.gov/geo/query/acc.cgi?acc=GSE231993.
