# Supplementary figures and images for: Gut microbiota metabolite indole-3-acetic acid maintains intestinal epithelial homeostasis through mucin sulfation
Source: Gut Microbes. 2024 Jul 27;16(1):2377576. doi: 10.1080/19490976.2024.2377576 (PMC11285290; doi:10.1080/19490976.2024.2377576)

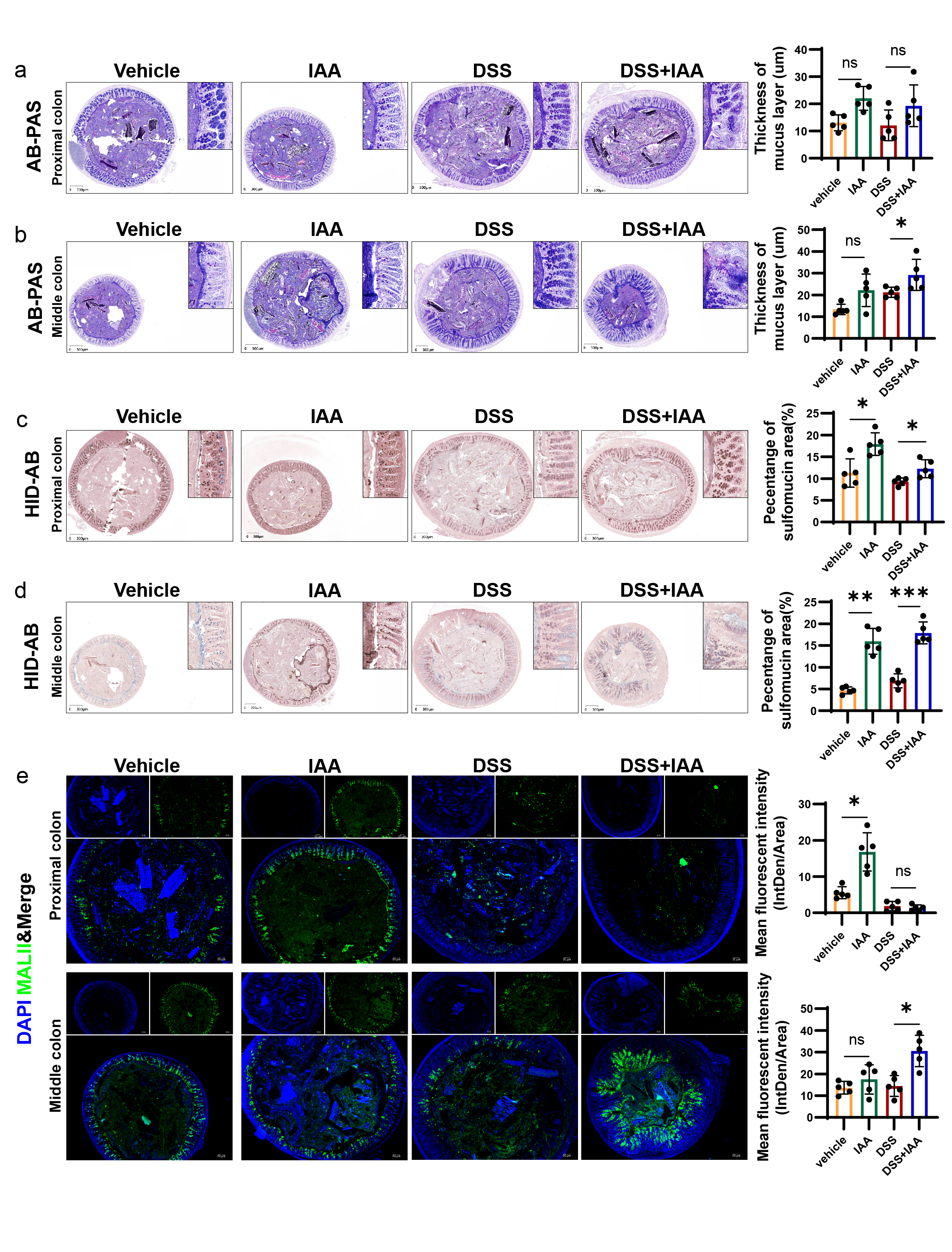

Supplement: Supplemental Material [file KGMI_A_2377576_SM1906.zip › Figure S1corrected.jpg]

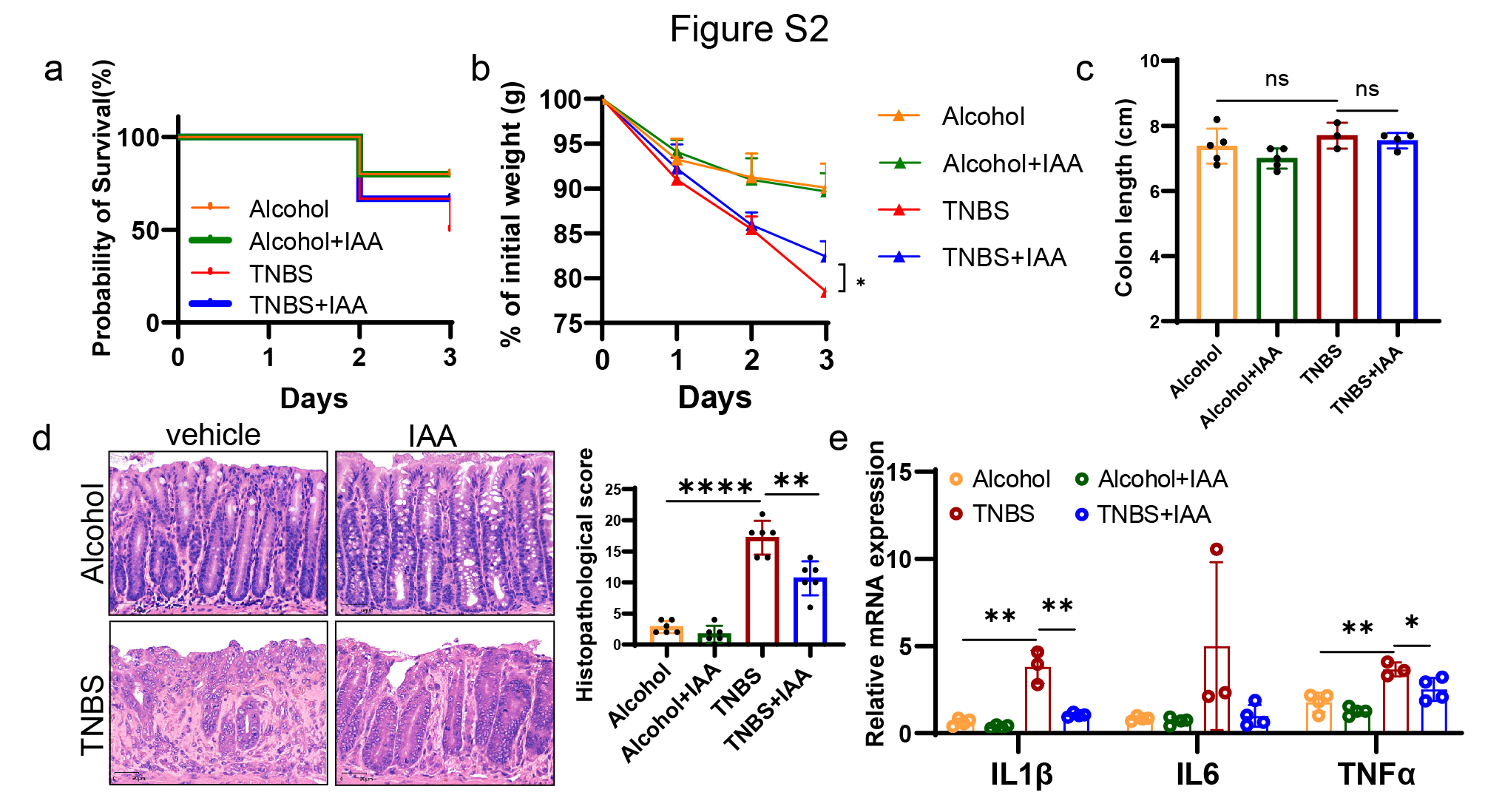

Supplement: Supplemental Material [file KGMI_A_2377576_SM1906.zip › Figure S2corrected.jpg]

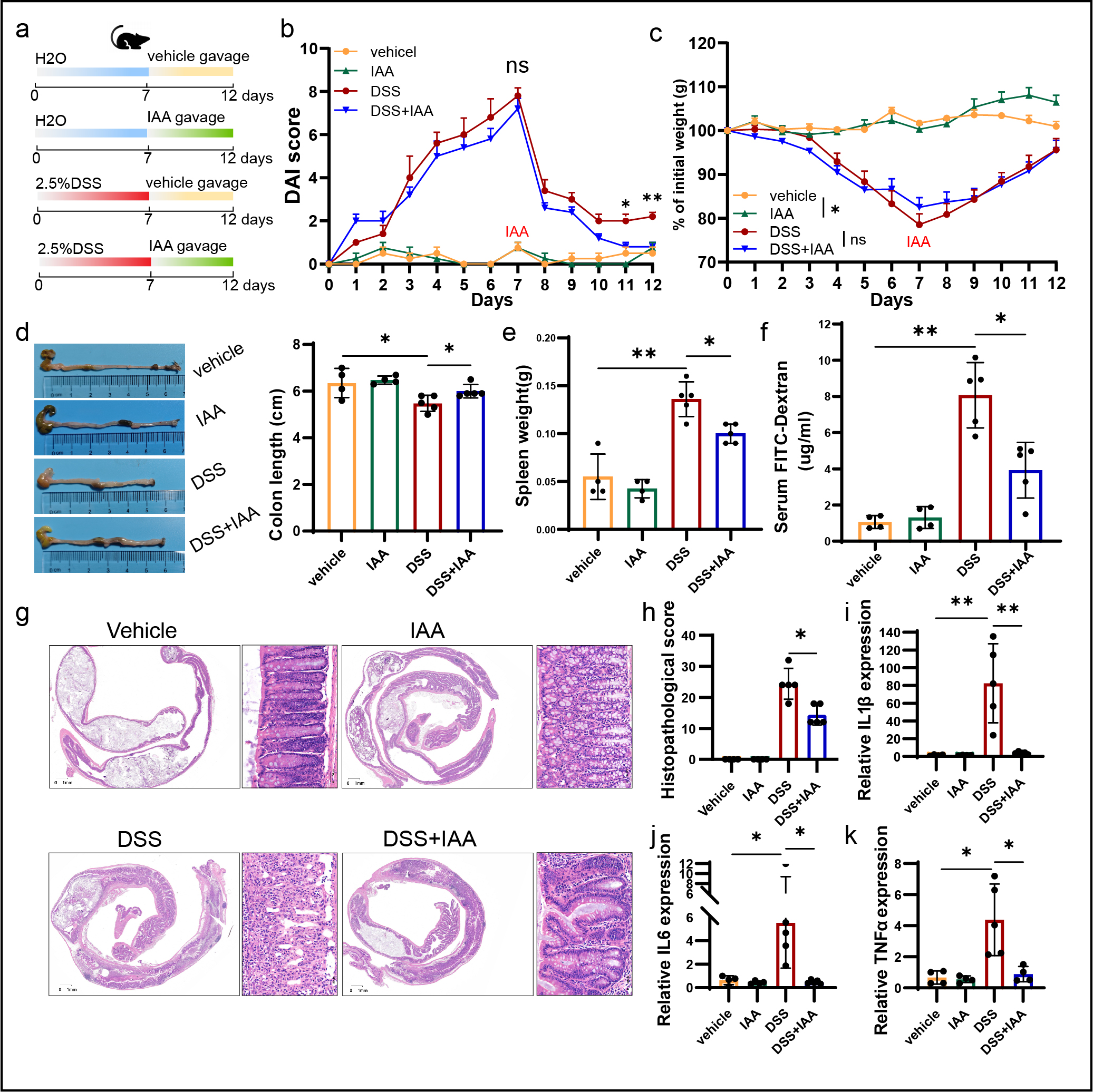

Supplement: Supplemental Material [file KGMI_A_2377576_SM1906.zip › Figure S3corrected.jpg]

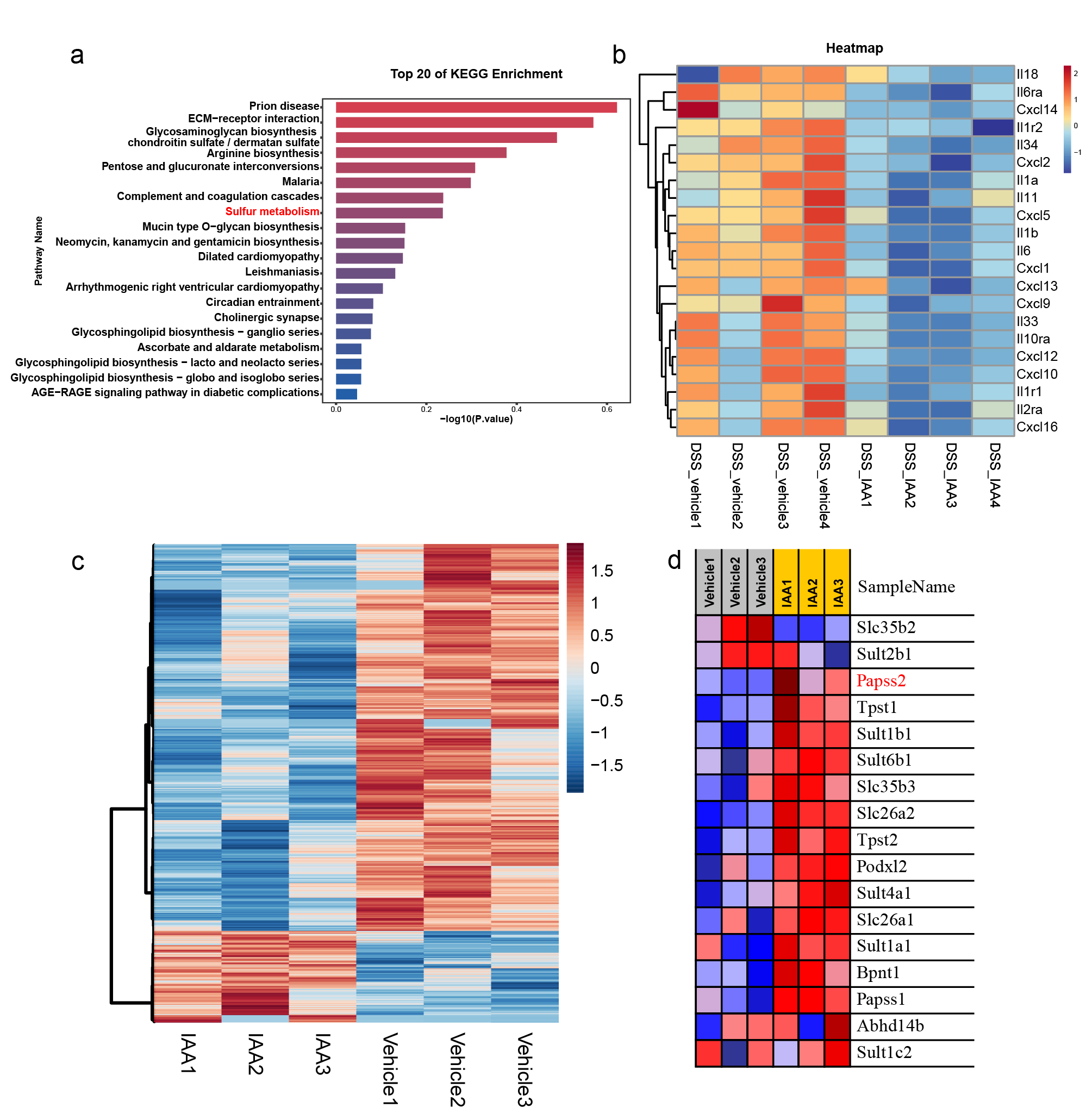

Supplement: Supplemental Material [file KGMI_A_2377576_SM1906.zip › Figure S4corrected.jpg]

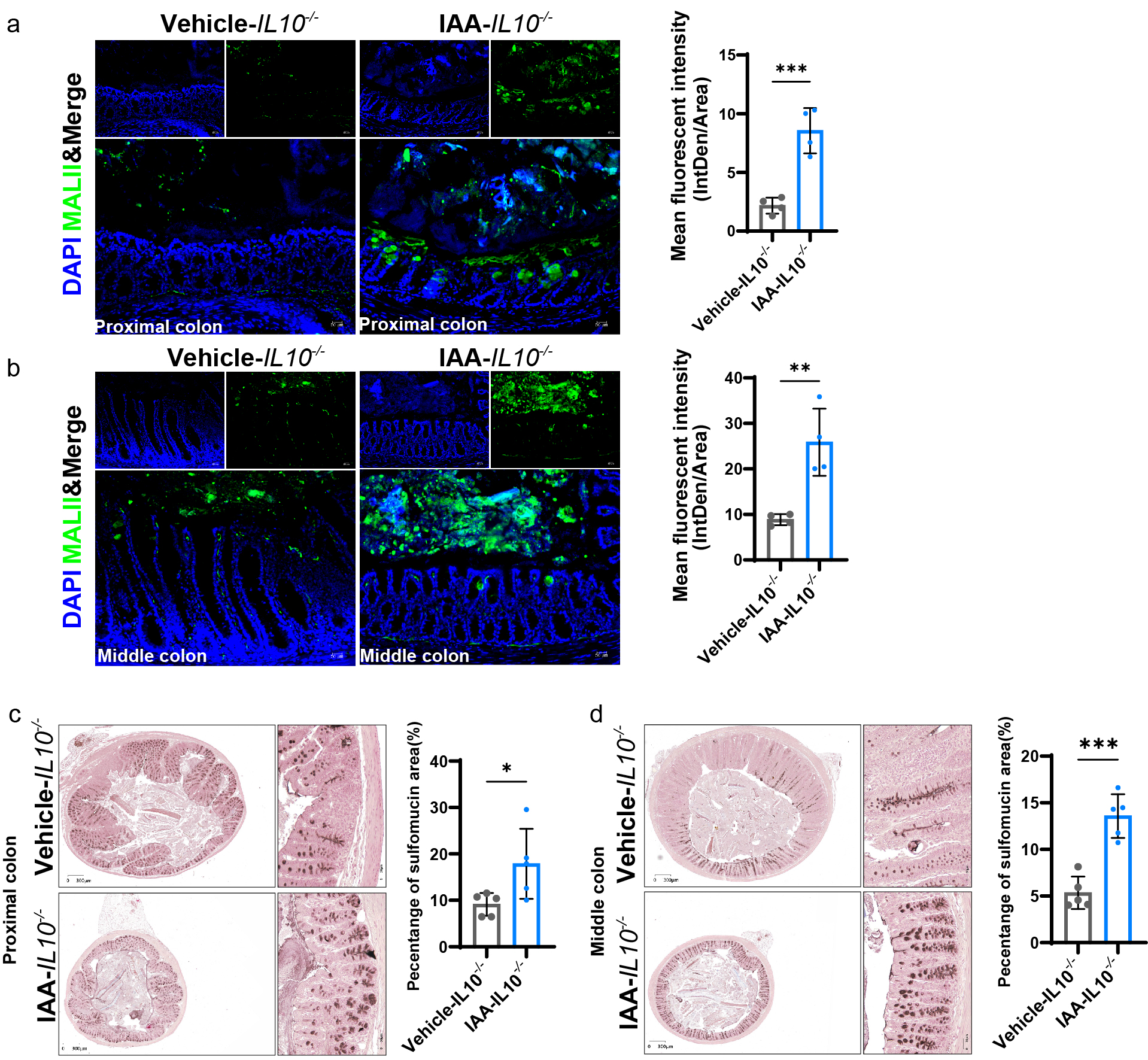

Supplement: Supplemental Material [file KGMI_A_2377576_SM1906.zip › Figure S5corrected.jpg]

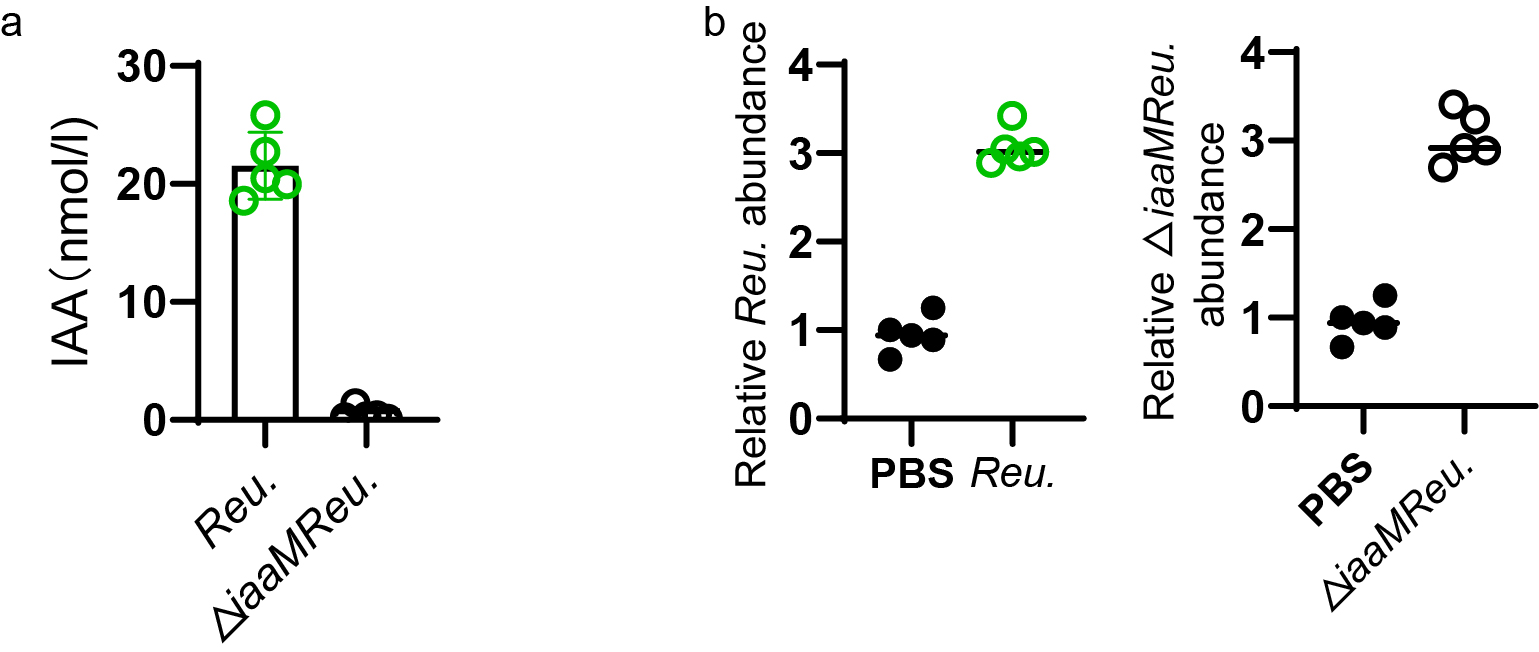

Supplement: Supplemental Material [file KGMI_A_2377576_SM1906.zip › Figure S6corrected.jpg]
