# Supplementary material for: Gut microbiota metabolite indole-3-acetic acid maintains intestinal epithelial homeostasis through mucin sulfation
Source: Gut Microbes. 2024 Jul 27;16(1):2377576. doi: 10.1080/19490976.2024.2377576 (PMC11285290; doi:10.1080/19490976.2024.2377576)
Supplement: SemiQuanttative Food Frequency questionnaire.docx [file KGMI_A_2377576_SM4753.docx]

**Dietary survey questionnaire**

I. General data of patients

1. Age _ Years-old Sex _

2. Education level: ① Illiterate or rarely literate□ ② elementary school□ ③ junior high school□ ④ senior high school/senior high school/secondary school□ ⑤ college/undergraduate□ ⑥ master 's degree and above□

3. What is the nature of your facility?

□ Public institution □ Company □ Self-employed/Private owner □ Others

4. Do you smoke:

Yes □ No □ Smoking frequency: daily ____ cigarettes

5. Did you drink alcohol:

Yes □ No □ Alcohol consumption frequency: 1 times a week □ 2-4 times a week □

5-6 times a week □

6 . Consumption of nutrient supplements/health food

Yes □ No □ "Yes" Please write the name of the health food: ___________________________

7 . Whether there are the following chronic non-communicable diseases:

Hypertension □ Hyperlipidemia □ Hyperglycemia □ Gout □ Constipation □ Tumor □ Others___________________________

If you have any of the above conditions, please specify when you were diagnosed: _Year_ Month

8 . Physical activity:

Usually with or without activity habits : Yes □ No □

Activity mode: mainly aerobic exercise □ mainly anaerobic exercise □ aerobic combined anaerobic exercise □ walking

II. Nutritional Assessment

Physical examination:

Height (cm): ____ Normal weight (kg): ____ Current weight (kg): _ BMI (kg/m ^2^): ____

(tick"√"at dominant hand) Handgrip strength (kg): left ____, right ____ upper arm circumference (cm): ____

Waist circumference (cm): ____ hip circumference (cm): ____ calf circumference (left, cm): ____

Body composition determination:

Total body fat (kg): Total lean body mass (kg): ____ Muscle mass (kg) Body fat percentage (%): ____

Limb skeletal muscle index (kg/m ^2^): ____ visceral fat area (cm ^2^): ____

III. Food Frequency Survey

1. Meal habits over the past week

| F1 | Meals over past week F2 | Meals at Restaurant in the past week F3 | Meals at Unit/School in the past week F4 |
| --- | --- | --- | --- |
| 1.Breakfast |  |  |  |
| 2. Lunch |  |  |  |
| 3. dinner |  |  |  |

2. Semi-quantitative Food Frequency Questionnaire (SQFFQ)

Remember if you have eaten the following foods in the past year and estimate the average amount and frequency of consumption of these foods, taboo foods hit X ()

| Food Name F5 | | Eat F6  1 No  2 Yes | Number of Meals (select one) | | | | Average consumption per consumption (g)  F11 |
| --- | --- | --- | --- | --- | --- | --- | --- |
|  |  |  | Times/Day  F7 | Times/Week  F8 | Times/month  F9 | Times/Year  F10 |  |
| Staple food | | | | | | | |
| 1 | Rice and products (rice/rice flour, etc.) (recorded as raw weight) |  |  |  |  |  |  |
| 2 | Wheat flour and products (steamed bread/noodles, etc.) (recorded as raw weight) |  |  |  |  |  |  |
| 3 | Other cereals and products (buckwheat/millet, etc.) (recorded as raw weight) |  |  |  |  |  |  |
| 4 | Fritters, oil cake |  |  |  |  |  |  |
| 5 | Other fried pasta (fried cake, tortilla, etc.) |  |  |  |  |  |  |
| 6 | Instant noodles |  |  |  |  |  |  |
| 7 | Potatoes (potatoes/taro/sweet potatoes, etc.) (recorded by raw weight) |  |  |  |  |  |  |
| 8 | Miscellaneous beans (mung bean/red bean/flower bean, etc.) (recorded as raw weight) |  |  |  |  |  |  |
| 9 | Corn flour (corn ballast, etc.) |  |  |  |  |  |  |
| Legumes | | | | | | | |
| 10 | Soybean (soybean/green bean/black bean, etc.) (dry weight) |  |  |  |  |  |  |
| 11 | Soybean milk |  |  |  |  |  |  |
| 12 | Tofu |  |  |  |  |  |  |
| 13 | Fermented bean curd (stinky tofu milk, bulky fermented bean curd, etc.) |  |  |  |  |  |  |
| 14 | Ready-to-eat soy products |  |  |  |  |  |  |
| 15 | Other soy products |  |  |  |  |  |  |
| 16 | Putrescent bamboo (including putrescent bamboo, oil peel, etc.) (dry weight) |  |  |  |  |  |  |
| Vegetables (recorded by raw weight) | | | | | | | |
| 17 | Fresh legume vegetables (lentils, beans, green beans, cowpeas, etc.) |  |  |  |  |  |  |
| 18 | Solanum vegetables (eggplant, tomato, green pepper, etc.) |  |  |  |  |  |  |
| 19 | Melon vegetables (cucumber, horn melon, zucchini, etc.) |  |  |  |  |  |  |
| 20 | Allium sativum (garlic seedlings, leeks, onions, etc.) |  |  |  |  |  |  |
| 21 | Stem vegetables (celery, lettuce, etc.) |  |  |  |  |  |  |
| 22 | Tuber roots (radish, lotus root, yam, etc.) |  |  |  |  |  |  |
| 23 | Brassica oleracea vegetables (cauliflower, kale, cabbage, etc.) |  |  |  |  |  |  |
| 24 | Leaf vegetables (spinach, rapeseed, pakchoi, cabbage, etc.) |  |  |  |  |  |  |
| 25 | Sauce pickled vegetables (bulk) |  |  |  |  |  |  |
| 26 | Sauce pickled vegetables (packaging) |  |  |  |  |  |  |
| Bacteria and algae | | | | | | | |
| 27 | Edible mushrooms Non-mushrooms (fungus, white fungus, etc.) (dry weight) |  |  |  |  |  |  |
| 28 | Mushrooms (Flammulina velutipes, Lentinula edodes, Pleurotus ostreatus, Volvariella volvacea, etc.) (fresh weight) |  |  |  |  |  |  |
| 29 | Porphyra (dry weight) |  |  |  |  |  |  |
| 30 | Kelp (fresh weight) |  |  |  |  |  |  |
| Fruits (recorded by edible weight) | | | | | | | |
| 31 | Citrus fruits (oranges, grapefruits, oranges, etc.) |  |  |  |  |  |  |
| 32 | Benevolent fruit (apple, pear, etc.) |  |  |  |  |  |  |
| 33 | Walnuts (peach, plum, pipa, dates, apricot, etc.) |  |  |  |  |  |  |
| 34 | Small fruits, berries (strawberries, grapes, macaque picks, etc.) |  |  |  |  |  |  |
| 35 | Tropical fruits (skin is not edible) (mango, banana, pineapple, etc.) |  |  |  |  |  |  |
| 36 | Tropical fruit (edible peel) (carambola, etc.) |  |  |  |  |  |  |
| 37 | Melon fruits (watermelon, melon, etc.) |  |  |  |  |  |  |
| Milk | | | | | | | |
| 38 | Whole milk |  |  |  |  |  |  |
| 39 | Low fat, skimmed liquid milk |  |  |  |  |  |  |
| 40 | Whole milk powder |  |  |  |  |  |  |
| 41 | Low-fat milk powder |  |  |  |  |  |  |
| 42 | Yogurt |  |  |  |  |  |  |
| 43 | Cheese |  |  |  |  |  |  |
| 44 | Ice cream |  |  |  |  |  |  |
| Meat (recorded by edible weight) | | | | | | | |
| 45 | Fresh (frozen) pork |  |  |  |  |  |  |
| 46 | Fresh (frozen) beef |  |  |  |  |  |  |
| 47 | Fresh (frozen) mutton |  |  |  |  |  |  |
| 48 | Fresh (frozen) poultry meat |  |  |  |  |  |  |
| 49 | Other fresh (frozen) meat (donkey, horse, pigeon, etc.) |  |  |  |  |  |  |
| 50 | Cooked pork |  |  |  |  |  |  |
| 51 | Cooked beef |  |  |  |  |  |  |
| 52 | Cooked mutton |  |  |  |  |  |  |
| 53 | Cooked poultry meat |  |  |  |  |  |  |
| 54 | Cooked other livestock meat (donkey meat, horse meat, pigeon meat, etc.) |  |  |  |  |  |  |
| 55 | Meat products (sausage, ham sausage, luncheon meat, etc.) |  |  |  |  |  |  |
| 56 | Pig liver |  |  |  |  |  |  |
| 57 | Pig kidney |  |  |  |  |  |  |
| 58 | Other animal viscera |  |  |  |  |  |  |
| Aquatic Products (Recorded as raw edible weight) | | | | | | | |
| 59 | Crucian carp |  |  |  |  |  |  |
| 60 | Silver carp |  |  |  |  |  |  |
| 61 | Grass carp |  |  |  |  |  |  |
| 62 | Tilapia |  |  |  |  |  |  |
| 63 | Other freshwater fish |  |  |  |  |  |  |
| 64 | Yellow Fish |  |  |  |  |  |  |
| 65 | Pomfret |  |  |  |  |  |  |
| 66 | Strap fish |  |  |  |  |  |  |
| 67 | Other marine fish |  |  |  |  |  |  |
| 68 | Shrimp |  |  |  |  |  |  |
| 69 | Crab |  |  |  |  |  |  |
| 70 | Mollusca (jellyfish, shellfish, snails, squid, etc.) |  |  |  |  |  |  |
| Egg | | | | | | | |
| 71 | Fresh eggs (eggs/duck eggs/quail eggs, etc.) |  |  |  |  |  |  |
| 72 | Salted duck egg |  |  |  |  |  |  |
| 73 | Preserved egg |  |  |  |  |  |  |
| Snacks, snacks | | | | | | | |
| 74 | Bread |  |  |  |  |  |  |
| 75 | Biscuits |  |  |  |  |  |  |
| 76 | Cream cake |  |  |  |  |  |  |
| 77 | Other pastries |  |  |  |  |  |  |
| 78 | White pumpkin seed |  |  |  |  |  |  |
| 79 | Peanut (recorded as edible weight) |  |  |  |  |  |  |
| 80 | Other nuts (walnuts, pistachios, hazelnuts, etc.)  (Record as weight of edible portion) |  |  |  |  |  |  |
| 81 | Chocolate |  |  |  |  |  |  |
| 82 | Fried small foods (chips, chips, etc.) |  |  |  |  |  |  |
| 83 | Puffed food |  |  |  |  |  |  |
| 84 | Candied fruit (bayberry, figs, dates, olives, kumquat and other preserves) |  |  |  |  |  |  |
| 85 | Cold fruit (Chen Pei Mei, rock sugar bayberry, etc.) |  |  |  |  |  |  |
| 86 | Preserved fruit (apple preserved, apricot preserved, plum dried preserved, mixed preserved, etc.) |  |  |  |  |  |  |
| 87 | Plum |  |  |  |  |  |  |
| 88 | Nine-system tangerine peel |  |  |  |  |  |  |
| 89 | Other plum classes (licorice olive, Chinese plum, Chinese apricot, nine system carambola) |  |  |  |  |  |  |
| 90 | Gudan class (Gudanpi, Citrus sinensis, Hawthorn dan) |  |  |  |  |  |  |
| 91 | Fruit cakes (hawthorn cake, hawthorn strips, jujube cake, etc.) |  |  |  |  |  |  |
| Beverages, condiments | | | | | | | |
| 92 | Carbonated beverage |  |  |  |  |  |  |
| 93 | Fresh fruit and vegetable juice |  |  |  |  |  |  |
| 94 | Fruit and vegetable juice drink |  |  |  |  |  |  |
| 95 | Lactobacillus beverage |  |  |  |  |  |  |
| 96 | Formulated Milk Beverage (Nutritional Express etc.) |  |  |  |  |  |  |
| 9 7 | Low calorie drinks |  |  |  |  |  |  |
| 98 | Tea drink |  |  |  |  |  |  |
| 99 | Milk tea |  |  |  |  |  |  |
| 1 00 | Coffee |  |  |  |  |  |  |
| 101 | Tea (without water) |  |  |  |  |  |  |
| 102 | Pepper products (dried, pepper flavored products/pepper noodles/canned fresh pepper, etc.) |  |  |  |  |  |  |

3. cooking oil and condiments

Please recall the consumption of cooking oil and condiments at your home over the past month and ask monthly questions on a household basis.

How many people do you usually eat together? ________People F12

|  | Edible Oil F13 | Consumption for Whole Family (grams) F14 |  |  | Condiment F13 | Consumption for Whole Family (grams) F14 |
| --- | --- | --- | --- | --- | --- | --- |
| 103 | Peanut oil |  |  | 112 | Common edible salt |  |
| 104 | Soybean oil |  |  | 113 | Low sodium salt |  |
| 105 | Canola oil |  |  | 114 | Soy sauce |  |
| 106 | Salad oil/blended oil |  |  | 115 | Vinegar |  |
| 107 | Sesame oil |  |  | 116 | Sugar |  |
| 108 | Animal oil |  |  | 117 | Sesame sauce |  |
| 109 | Tea oil, olive oil |  |  | 118 | Sauce (yellow sauce, bean paste, etc.) |  |
| 110 | Corn oil, sunflower seed oil |  |  | 119 | Chicken essence, MSG, chicken powder, etc. |  |
| 111 | Other oil |  |  | 120 | Other condiments (please write the name) |  |

Investigator 's signature:

Date:
